# On-chip generation of Bessel–Gaussian beam via concentrically distributed grating arrays for long-range sensing

**DOI:** 10.1038/s41377-023-01133-2

**Published:** 2023-04-14

**Authors:** Zihao Zhi, Quanxin Na, Qijie Xie, Baisong Chen, Yingzhi Li, Xiaobin Liu, Xuetong Li, Lijun Wang, Guoqiang Lo, Junfeng Song

**Affiliations:** 1 State Key Laboratory on Integrated Optoelectronics, College of Electronic Science and Engineering, Jilin University, Changchun, 130012 China; 2 Peng Cheng Laboratory, Shenzhen, 518000 China; 3 State Key Laboratory of Luminescence and Application, Changchun Institute of Optics, Fine Mechanics and Physics, Chinese Academy of Sciences, Changchun, 130033 China; 4 Advance Micro Foundry Pte. Ltd., 11 Science Park Road, Science Park II, 117685 Singapore

**Keywords:** Silicon photonics, Imaging and sensing, Photonic devices

## Abstract

Bessel beam featured with self-healing is essential to the optical sensing applications in the obstacle scattering environment. Integrated on-chip generation of the Bessel beam outperforms the conventional structure by small size, robustness, and alignment-free scheme. However, the maximum propagation distance (Z_max_) provided by the existing approaches cannot support long-range sensing, and thus, it restricts its potential applications. In this work, we propose an integrated silicon photonic chip with unique structures featured with concentrically distributed grating arrays to generate the Bessel–Gaussian beam with a long propagation distance. The spot with the Bessel function profile is measured at 10.24 m without optical lenses, and the photonic chip’s operation wavelength can be continuously performed from 1500 to 1630 nm. To demonstrate the functionality of the generated Bessel–Gaussian beam, we also experimentally measure the rotation speeds of a spinning object via the rotational Doppler Effect and the distance through the phase laser ranging principle. The maximum error of the rotation speed in this experiment is measured to be 0.05%, indicating the minimum error in the current reports. By the compact size, low cost, and mass production potential of the integrated process, our approach is promising to readily enable the Bessel–Gaussian beam in widespread optical communication and micro-manipulation applications.

## Introduction

The Bessel beam, with a significant depth of field and self-healing characteristics^1^, has been applied in widespread applications, including quantum entanglement^2^, underwater 3D imaging^3^, optical micromanipulation^4^, microscope^5^, and so on. There have been various ways to generate a Bessel beam, such as circular slit and lens^6^, axicon^7,8^, and spatial light modulator (SLM)^9^. However, these methods are complicated due to the usage of bulky optical elements. It hinders the Bessel beam generation system from being applied in practical applications. Recently, several compact systems have been proposed to generate Bessel beams by using photonic integrated circuits (PICs)^10^, metasurfaces^11,12^, integrated waveguide^13^, and 3D-printed fiber^14^. The methods based on PICs only generate a quasi-1D Bessel beam. The metasurfaces-based systems require accurate alignment and thus occur instability issues. The technique relied on the 3D-printed fiber cannot effectively manipulate the polarization of incident beams. Moreover, the propagation distance of Bessel beams generated by the above technologies is short (detailed comparison illustrated in Supplementary Table S1 and Supplementary Section 1), which is far from the theoretically calculated infinite distance of the Bessel beams. It significantly restricts the applications of the Bessel beam in scenarios requiring long propagation distances, such as optical sensing, optical communication, and so on.

The Bessel beam superposed by plane waves exhibits ideal properties of infinite extension and infinite energy. However, the practical generation of the Bessel beam deviates from the ideal beam due to the maximum propagation distance length of Z_max_^6^. It is attributed to not only the plane wavelet’s finite extension but also the wavelet’s short superposition area. The Bessel–Gaussian beam^15^ (BGb) is the solution of the paraxial wave equation and can be obtained by the superposition of a series of Gaussian beams. It carries finite power and can be transformed into the Bessel beam through transverse modulation. Most importantly, the Bessel beam and BGb share the same intensity profile in the form of the Bessel function at a certain propagation distance. In theory, the BGb can also propagate infinitely^16^. However, this infinity property has yet to attract much attention due to the similar propagation characteristic of the zero-order BGb to that of the ordinary Gaussian beam. For high-order BGb, this infinite propagation characteristic can significantly benefit the study of the Bessel beam through collimation technology. A detailed comparison of these two beams and the principle of the infinite BGb can be found in Supplementary Fig. S1.

Thanks to the compactness, high stability, and compatibility with the complementary metal oxide semiconductor (CMOS) process, the use of integrated photonic chips to regulate the optical field has been developed rapidly in recent years^17–20^. It is worth noting that the vortex beam emitters are different from the BGb generator in terms of the spiral distribution of the wavefront. Specifically, the near-field beam generated by the vortex beam emitters is not a BGb, even though the beam can carry orbital angular momentum (OAM). The beam with single ring profile, referred to Supplementary Fig. S1d can be used to carry the OAM if the phase of these wavelets is helically stacked to 2*πl*, where *l* is the topological charge. In addition, the existing integrated chips^10,13^ for Bessel beams generation are difficult to be applied in practice due to their short propagation distance. Therefore, an integrated photonic chip that can generate BGb and work for a long distance is essential to the application of Bessel beams.

Rotation is a fundamental phenomenon in nature, existing in a form of molecular spin^21^ and micro-particle spin^22^ in a microcosm, as a mechanical device rotation^23^ the macroscopic world and even in the rotating black holes^24^ of the universe. An effective approach to measure rotation speed is essential to reveal physics characteristics, manage precise machinery and analyze the composition of celestial bodies. The on-chip Bessel–Gaussian beam can provide an integrated solution for effective rotation measurement.

In this work, we propose an unprecedented structure based on silicon photonic grating arrays to generate the Bessel Gaussian beam with a long propagation distance. The grating arrays are concentrically distributed on the chip. In the experiment, we applied the chip to generate an infinite first-order Bessel–Gaussian vector beam with azimuthal polarization. Moreover, the BGb profiles operated at wavelength range from 1500 nm to 1630 nm are obtained. The detailed beam profiles can be referred to Supplementary Figs. S8–S10. The light intensity distribution along the vertical cut of the BGb profiles are also obtained. The spatial distribution of the light intensity follows the first kind of Bessel function. The detail can be found in Supplementary Fig. S11. Last but not least, we also applied the azimuthally polarized BGb to measure rotation speed and distance of a target simultaneously.

## Results

According to our previous work^25^, the electric field of the Bessel beam can be obtained from the superposition of multiple diversely polarized wavelets with the same initial phase. Based on the Cobweb structure discussed in ref. ^25^, the electric field of the generated Bessel beam can be expressed respectively by

1 $$\vec E_B\left( {\rho ,\phi ,z} \right) = A_BJ_1\left( {k_\rho \rho } \right)\left( {\begin{array}{*{20}{c}} {\sin \phi } \\ { - \cos \phi } \end{array}} \right)$$

Where (*ρ*, *ϕ*, *z*) is a point in the cylindrical coordinate system. *A*_*B*_ is the constant amplitude for a given *z*, which does not affect the profile of the Bessel beam, *J*_1_ (*x*) is the first kind of Bessel function, and *k*_*ρ*_ is the projection of the wavenumber on the direction. However, the above formulas lack of important information about the measurable length. The BGb is obtained by the superposition of a series of Gaussian beams and the evolution process of the BGb can be described by Supplementary Eq. (S3.5). There are two angles are worthy of note, which are cone half-angle, called emission angle in optical phased arrays (OPAs), and divergence angle of Gaussian beam. By comparing the emission angle (*θ*_G1_) and the divergent half-angle (*θ*_G2_) of the Gaussian wavelets, we can get four cases of measurable length, as shown in Supplementary Fig. S2 and Supplementary Table S2. Noted that the BGb suitable for long-distance propagation can be obtained only in the case of *θ*_G2_ ≥ |*θ*_G1_|.

**Fig. 1:**
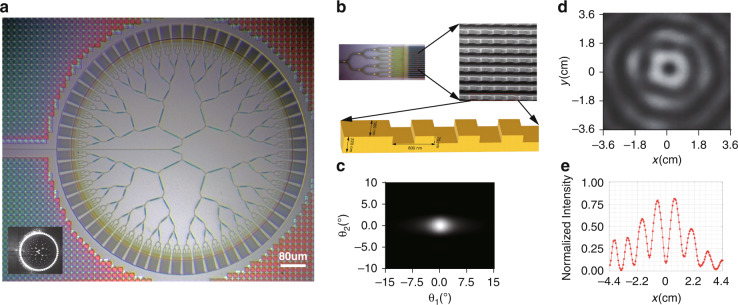
On-chip Bessel–Gaussian beam generator. **a** Optical microscope image for an integrated photonic chip used as a Bessel–Gaussian beam generator. The inset is a surface image captured when the laser light emitted from the chip. **b** Enlarged view of local grating arrays in the photonic chip and Scanning electron microscope (SEM) image of the grating arrays. **c** Simulated intensity distribution of far-field from the grating arrays. **d** Experimentally measured BGb profile at 5.91 m. **e** One-dimensional intensity distribution of light field at 5.91 m

Based on the above derivation, the integrated photonic chip is designed as illustrated in Fig. 1a. The whole ring structure has a diameter of 870 μm and is fabricated on a silicon-on-insulator (SOI) substrate by the Singapore Advanced Micro Foundry (AMF) standard process. The structure is crucial to the generation of infinite BGb, and thus the detailed description is made in Supplementary Section 4 (Design of waveguide structure). The integrated photonic chip consists of a spot mode coupler, multiple Y beam splitters, and 64-channel grating emitters arranged circularly. The laser is coupled into the bus waveguide (500 nm × 220 nm) through a lens fiber. A polarization controller (PC) was used to align the light to quasi-TE mode of the waveguide. Subsequently, it is coupled to the 64-channel grating emitters through the Y beam splitters. The grating array’s structure is illustrated in Fig. 1b. In grating-based antenna areas, there are 16 parallel waveguides (380 nm × 220 nm), and the spacing between adjacent waveguides is 1.62 μm, the grating period is 800 nm, the duty ratio is 0.5, and the etching depth is 70 nm. The aperture of the antenna area is 30.38 μm × 40 μm. Thanks to the symmetric structure of the Y beam splitter and the concentrically distributed grating arrays emitter called OPAs^10^, the phase of light emitted from 64 channel grating array is the same. After the light comes out of the grating arrays, Gaussian-like light spot is formed in the far field, as shown in Fig. 1c (Simulation details in Supplementary Section 3). A BGb profile at 5.91 m is formed by coherent superposition of 64 circularly distributed Gaussian-like light beams operated at 1630 nm, as shown in Fig. 1d. The corresponding one-dimensional intensity distribution follows the first-kind Bessel function as shown in Fig. 1e.

### BGb generated by the integrated photonic chip

We used the grating arrays-based silicon chip to generate infinity BGb. Without aided by any optical lens, the measured spot diameter of the innermost ring is 0.41 cm, 1.54 cm, and 2.45 cm at *z* = 1.55 m, 5.91 m, and 10.24 m, respectively (see from Supplementary Figs. S8–S12). The measured results show the spot diameter increases as the propagation distance, indicating the BGb propagates in a conically divergent manner. Moreover, the far-field spot profile can be maintained during the wavelength of the incident beam changed from 1500 nm to 1630 nm continuously, which exhibits potentially compatible with the wavelength division multiplexing systems. The capabilities of the long propagation distance and broad operating wavelength range benefit from the unique chip structure and design. Hence, the condition of *θ*_G2_ ≥ |*θ*_G1_| for infinite propagation distance can be maintained over the 130-nm bandwidth. All of operating wavelength from 1500 nm to 1630 nm can support the generated BGb to spread to infinity. Furthermore, the existence length of BGb is still measurable but suffers from limitation, when the wavelength of the incident light increases beyond 1630 nm. The detail can be found in Supplementary Fig. S7. In addition, we studied the self-healing characteristic of the BGb in both the transverse and longitudinal directions. In the experiment, we record the profile of the spot as the copper wire moves in the direction perpendicular to the propagation. The shape of the obstacle can no longer be observed in the beam profile after a certain propagation distance. It indicates the self-healing characteristic of the BGb. The detail can be found in Supplementary Fig. S13 in the Supplementary Material. In the longitudinal case, a specific size obstacle does not affect the contour of the innermost ring (see Supplementary Video 1 (SV1)).

**Fig. 2:**
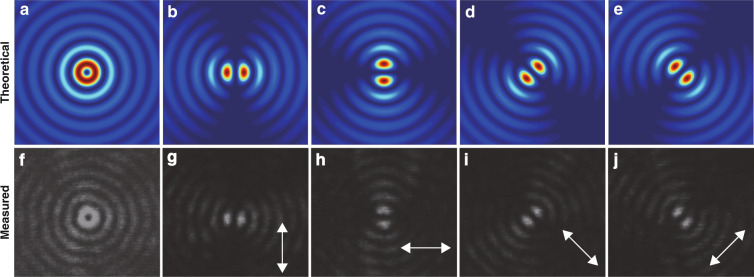
The polarization characteristics of BGb generated by the integrated photonic chip. **a** The far-field light intensity distribution, **b**–**e** under different polarization states, theoretically. **f** Measured intensity distribution at a distance of 2.2 m from the chip surface, **g**–**j** after a polarizer in the directions indicated by the arrows

The polarization characteristics of BGb generated by the integrated photonic chip are entirely different from those of Bessel beams in the previous works^6–14^ (Supplementary Table S1). Here, a first-order vector beam with polarization singularity centered at the optical field is generated. As shown in Fig. 2, the experimental measurement (second row) perfectly matches with the theoretical results (first row), and the simulation details can be seen in Supplementary Section 3. The intensity distribution of BGb rotates with the rotation of the linear polarizer. The dynamic polarization change can be seen in Supplementary Video 2 (SV2). The BGb with the azimuthal polarization is very useful in tight focusing system^26,27^ and optical communications^28^.

### Measurement of rotation speed and distance of a spinning object via using the on-chip generated BGb

The rotation speed measurement based on the rotational Doppler Effect has attracted attention. However, the existing methods rely on the light beam with OAM generated by SLM^29–31^. It inevitably increases the complexity of using bulky detection systems. The integrated generation of the Bessel beam can provide a simple structure for effectively detecting light beams blocked by obstacles. However, the previously reported Bessel beam generator^10–14^ cannot be used in long-distance scenes because of its short propagation distance. So far, there have seldom been reports on the rotation-speed measurement using the BGb generated on a chip. Owing to the BGb generated by our chip can be easily measured beyond 10 m (Supplementary Section 5), the measurement of this speed is no longer a problem.

**Fig. 3:**
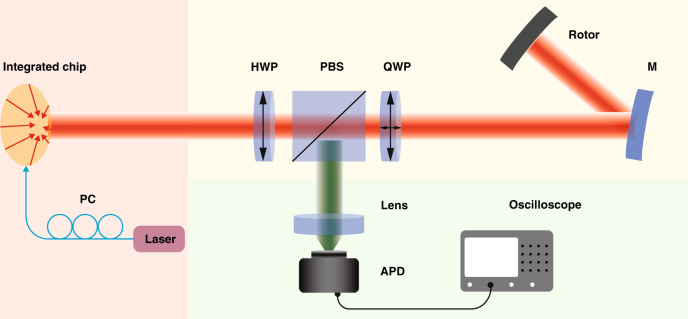
Schematic diagram for experimental setup of rotation-speed measurement of a spinning object using the BGb generated by the Grating arrays-based photonic chip. It consists of three sections. Source section: the tunable laser passes through the polarization controller (PC), lens fiber, and enters the chip to generate the BGb; Transmission section: BGb passes through half-wave plate (HWP), polarization beam splitter (PBS), quarter wave plate (QWP) sequentially, and is irradiated onto the surface of a rotating object through a mirror; Detection section: the echo with information enters Avalanche photodiodes (APD) through a plan convex lens with a focal length of 30 mm and finally enters the oscilloscope. The distance between the chip and the mirror is 1.4 m, and the rotor is an optical chopper without blades of which rotation speed can be accurately controlled

It is worth noting that the detection method differs from the previously reported methods^29,32^. The azimuthally polarized beam is emitted from the grating arrays-based silicon chip. One of the polarization states of the light beam is selected through a polarizer. The beam can be seen as the superposition of two OAM modes of opposite topological charges, *l* = ±1. This new method has a more straightforward structure and makes the detection system handy. As shown in Fig. 3, the generated BGb passes through the half-wave plate (HWP) to change the polarization direction of polarized light. Subsequently, the polarized BGb is divided into two orthogonal linearly-polarized beams by a polarization beam splitter (PBS). Next, a quarter-wave plate (QWP) is used to change the linearly polarized light into circularly polarized light or elliptically polarized light. HWP and PBS together form a linear polarizer to control the polarization direction, and PBS and QWP together form a spatial circulator to receive echoes in long-distance sensing. The reflected light passes through the mirror, QWP, and PBS successively and is detected by the Avalanche photodiodes. The frequency shift information can be extracted through the oscilloscope. For the first-order BGb, *f*_mod_ = Ω/π and Ω is the angular frequency of rotation, we have strictly proved the method in Supplementary Section 6.

**Fig. 4:**
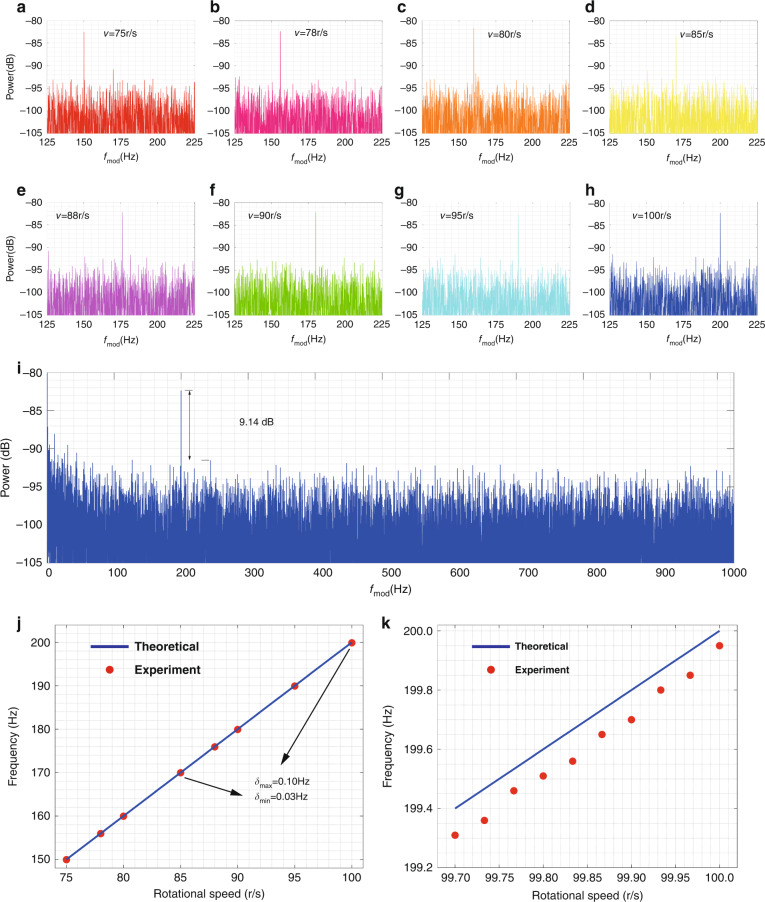
Measurement results of rotating speed using BGb generated on-chip. **a**–**h** The observed power spectrum at the indicated rotational speed over a data collection period of 20 s. **i** Detailed power spectrum with 0–1000 Hz at 100 r/s. **j** The measured results at different rotating speeds (75–100 r/s). **k** The velocity resolution measurement, there are ten rotational speeds at a step of 0.033 r/s from 99.7 r/s to 100 r/s. For **j** and **k**, the red points are the measured data, and the solid blue lines are the theoretical value

We process the recorded temporal waveform via the Fast Fourier Transform (FFT) to obtain the power spectrum. The sample duration of the waveform shows an impact on the measurement accuracy. To balance the measurement accuracy and time consumption, we adopted the FFT of signal waveform within 20 s to measure the rotation speed. The comparison among different sample durations can be seen in Supplementary Fig. S14 of the Supplementary Material. Figure 4a–h shows the power spectra of the corresponding velocities. The signal-to-noise pedestal ratio (SPNR)^33^ is greater than 9 dB in Fig. 4i, which means that the target signal can be extracted more easily. The dynamic process of the test can be seen from Supplementary Video 3 (SV3). For the measurement of different rotational speeds (75–100 r/s), the experiment results agree well with theoretical predictions, as shown in Fig. 4j. The maximum absolute error is 0.1 Hz, corresponding to the maximum relative error is 0.05%. Further, we investigated the velocity resolution of this system. In the range of 99.7–100 r/s, we measured ten velocities at a step of 0.033 r/s. The results in Fig. 4k indicate that the measured frequency shift increases linearly with the rotational speeds. The discrepancy between the measured results and the theoretical calculation is less than 0.1 Hz, and the accuracy of velocity measurement can be further improved by calibration.

**Fig. 5:**
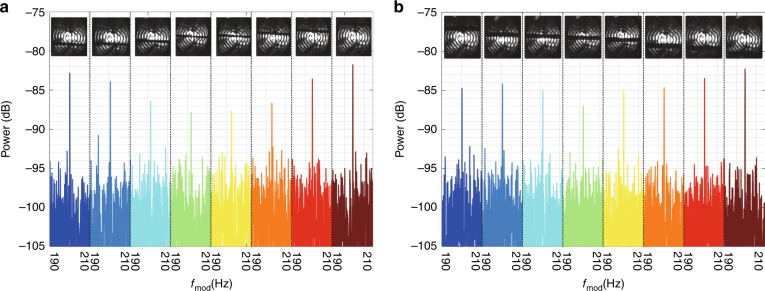
The rotation speed measurement with obstacles. **a** The obstacles are cylindrical copper wire with a diameter of 1.18 mm and **b** 2 mm. They are 0.801 m away from the chip surface, and the rotation speed is 100 r/s. The power spectral line in each cell is measured under the light spot condition above the cell

Furthermore, we studied the influence of an obstacle on the rotational speed measurement. The demonstration for obstacle movement along the direction perpendicular to the light beam propagation^34^ has been reported. We put a copper wire behind the QWP and moved its position from down to up (or up to down in Fig. 5b) to change the beam blocking position in Supplementary Video 4 (SV4, The dynamic process of the test with an obstacle, which is a 2 mm diameter copper wire). When the position of the copper wire is fixed in the spot, we use the incomplete spot to measure the rotation speed. As shown in Fig. 5, the peak value of the measured power spectrum decreases as the obstacle moves from one edge changes to the center and then increases again as the obstacle moves from the center to the other edge. The peak power of the beating signal changed as the obstacle position exhibited a “V” shape trend. The results show that the closer the obstacle to the center, the greater the influence. The phenomenon is similar to that in the reported work, in which power loss of the beat signal increases as the obstacle gets farther away from the SLM surface^34^. The comparison between Fig. 5a and Fig. 5b shows larger obstacles exhibit a severer influence on the measurement results (green lines: −86.99 dB in Fig. 5a and −87.85 dB in Fig. 5b). It shows that the BGb produced by the chip has a unique anti-interference capability due to its self-healing characteristics of BGb.

To our best knowledge, it is the first demonstration of rotation speed measurement by using the BGb generated by the Grating arrays-based photonic chip. The approach outperforms the SLM-base method by the simple light source configuration. In addition, we have obtained the minimum measurement error compared to the reported works^29,34–40^ (Supplementary Table S3 in Section 9). Finally, we investigated the effect of the obstacle moving perpendicular to the direction of propagation on the rotational velocity measurement and obtained the result that the peak value of the measured power spectrum changes in a “V” shape. The result provides a powerful experimental data reference for studying the self-healing behavior of BGb in three-dimensional media.

**Fig. 6:**
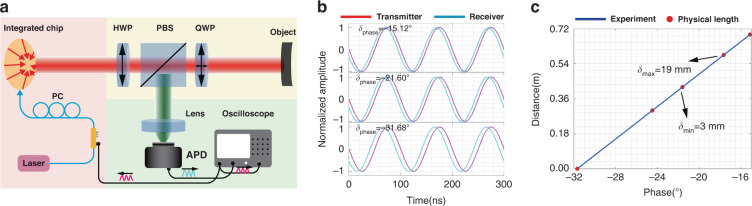
Phase laser ranging results. **a** Schematic diagram of distance measurement using phase ranging principle. **b** The phase difference between the transmitted and received signals at different target distances. **c** The distance obtained by the phase laser ranging method (solid blue lines) and physical length (red points)

Last but not least, we also measure the distance information of a rotational object by using the principle of phase ranging^41^. We added a lithium niobate (LiNbO_3_) intensity modulator prior to the PC as in Fig. 6a. The function generator embedded in an oscilloscope is used to generate a 10-MHz sinusoidal signal (magenta line in Fig. 6b) for driving an intensity modulator. At this time, the light emitted from the photonic chip is modulated. The BGb is reflected by the target and detected by APD to form the received signal (cyan line in Fig. 6b). Two signals are recorded by the oscilloscope using the average sampling mode. The distance information can be obtained by measuring the phase difference between the above two signals. As is shown in Fig. 6c, the measured distance is consistent with the actual distance. The maximum error is 19 mm, and the minimum is 3 mm. To our best knowledge, we are the first to demonstrate the simultaneous measurement of a spinning object rotation speed and distance by using the BGb generated from the grating arrays-based photonic chip.

## Discussion

The infinite Bessel–Gaussian beam emitter is designed and fabricated using the grating arrays-based photonic chip. The chip can be operated in the wavelength range of 1500–1630 nm. To the best of our knowledge, this is the first integrated photonic chip that can emit two-dimensional BGb and detect the light spot of the Bessel function distribution at 10.24-m away. The BGb is an azimuthally polarized beam with many potential applications in free-space optical communication.

For the first time, we use the generated BGb to measure the rotation speed and distance simultaneously. The experimental results are consistent with the theoretical or actual values, and the minimum error (0.05%) reported present is obtained. Furthermore, this method’s velocity resolution and robustness have been studied by measuring small rotational speed changes and large-diameter copper wire occlusion. We found that when the obstacle moves from edge to center and then to another edge, the peak value of the power spectrum changes in a “V” shape. This excellent anti-interference capability is expected to be applied in optical communication in complicated environments.

The accuracy of the measured beam profile, rotation speed, and distance can be further improved through subsequent optimization of the chip, including improving the directivity of Grating arrays and reducing the loss of Y beam splitters. The grand goal of photonic chips is to solve complex tasks on a single chip^42^. Therefore, it is the development trend in the future to add modulation modules to each grating array, i.e., optical phased arrays, make each light emitting unit programmable, and integrate light sources and detectors on the same chip. This extraordinary chip can realize advanced research and applications related to Bessel–Gaussian beams, such as optical communication and optical sensing.

## Materials and methods

### Simulation method

Simulation results and image processing were done by using MATLAB (Math Works, USA) and the simulation principle and parameters are detailed in Supplementary Section S3. The waveguide structure design of the integrated photonic chip is completed by FDTD (ANSYS Lumerical, USA). The simulation details can be seen in Supplementary Section S4.

### Measurement method

The far-field spot profiles of the Bessel–Gaussian beam were measured by using lasers (TSL-550-CL-Band, Santec, Japan) and infrared camera (Bobcat 640 GigE, Xenics, Belgium). The rotation speed and distance are measured by using the Avalanche photodiodes (APD410C, Thorlabs, USA) and oscilloscope (MSO8064, RIGOL, China).

## Supplementary information

Supplementary Information for On-Chip Generation of Bessel-Gaussian Beam via Concentrically-Distributed Grating Arrays for Long-Range Sensing

The longitudinal self-healing characteristic of the Bessel Gaussian Beam generated on chip

The intensity distribution of the Bessel Gaussian Beam generated by integrated photonic chip after passing through rotating polarizer

Animation when measuring the speed of rotating objects

Animation of rotational speed measurement with an obstacle
