# Supplementary material for: On-chip generation of Bessel–Gaussian beam via concentrically distributed grating arrays for long-range sensing
Source: Light Sci Appl. 2023 Apr 14;12:92. doi: 10.1038/s41377-023-01133-2 (PMC10102187; doi:10.1038/s41377-023-01133-2)
Supplement: Supplementary file 1 — Supplementary Information for On-Chip Generation of Bessel-Gaussian Beam via Concentrically-Distributed Grating Arrays for Long-Range Sensing [file 41377_2023_1133_MOESM1_ESM.docx]

Supplementary Information for

**On-Chip Generation of Bessel-Gaussian Beam via Concentrically-Distributed Grating Arrays for Long-Range Sensing**

Zihao Zhi^1^, Quanxin Na^2^, Qijie Xie^2^, Baisong Chen^1^, Yingzhi Li^1^, Xiaobin Liu^1^, Xuetong Li^1^, Lijun Wang^2,3^, Guoqiang Lo^4^, and Junfeng Song^1, 2^*,

*Corresponding author. Email: songjf@jlu.edu.cn;

**Affiliations:**

^1^State Key Laboratory on Integrated Optoelectronics, College of Electronic Science and Engineering, Jilin University, 130012 Changchun, China.

^2^Peng Cheng Laboratory, 518000 Shenzhen, China.

^3^State Key laboratory of Luminescence and Application, Changchun Institute of Optics, Fine Mechanics and Physics, Chinese Academy of Sciences, 130033 Changchun, China.

^4^Advance Micro Foundry Pte. Ltd., 11 Science Park Road, Science Park II, 117685, Singapore.

**This file includes:**

Supplementary Section 1 to Section 9

Tables S1 to S3

Figures S1 to S14

Supplementary video caption V1 to V4

References (1 to 22)

**Other Supplementary Materials for this manuscript include the following:**

Supplementary video V1 to V4

Section 1. Research on Bessel Beam Emitters in Recent Years

**Table S1. Progress and comparison of Bessel beam emitters in recent years**

| Year | Length | polarization | Wavelength | Size | Image |
| --- | --- | --- | --- | --- | --- |
| 2017^1^ | < 90 μm  (Fig. S4,  Fig. 2f) | circularly polarized | 532-800 nm | >4 mm  (Fig. S2) | 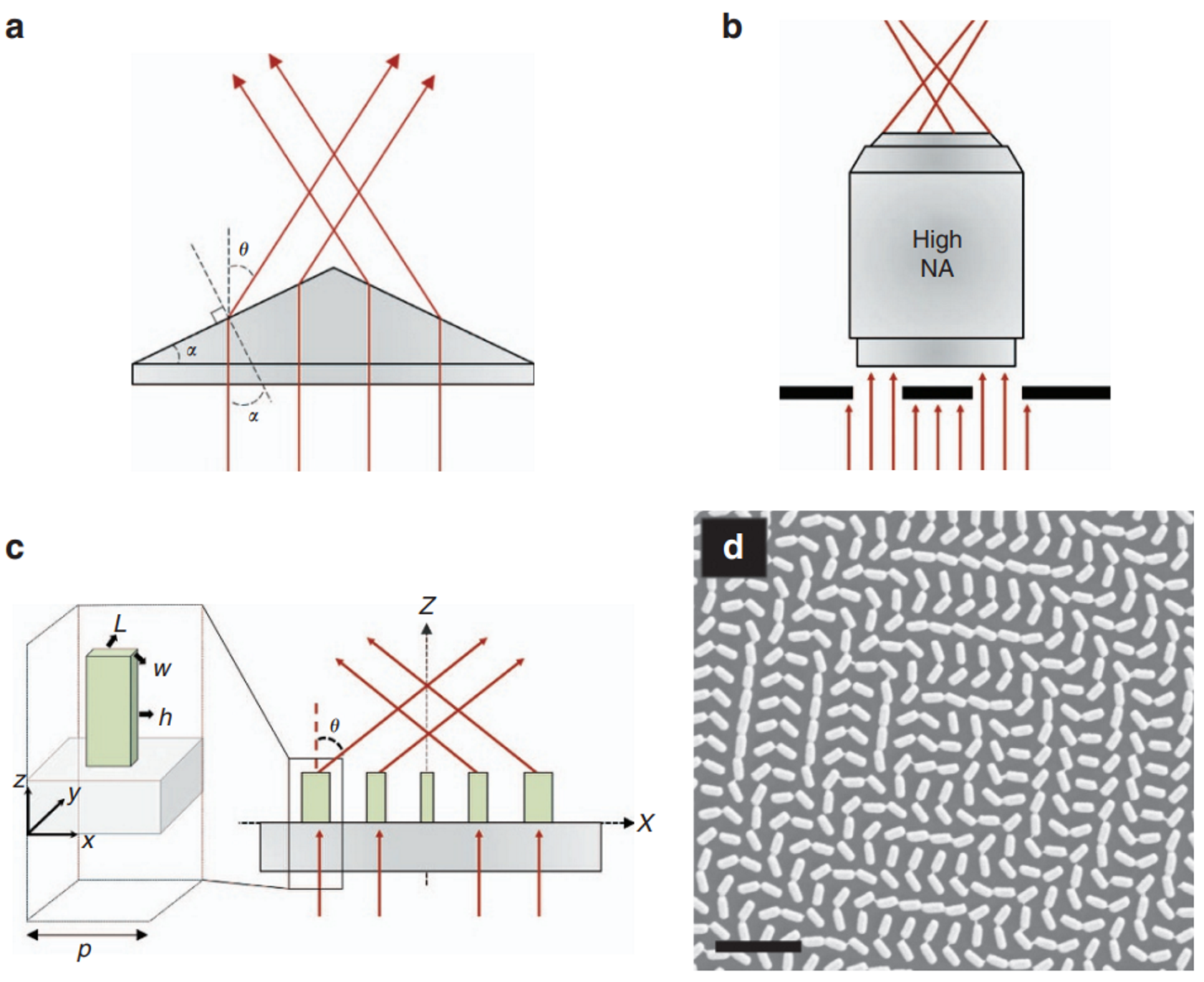 |
| 2017^2^ | 14 mm | -- | 1550 nm | 0.64 mm × 0.65 mm  (Antennas) | 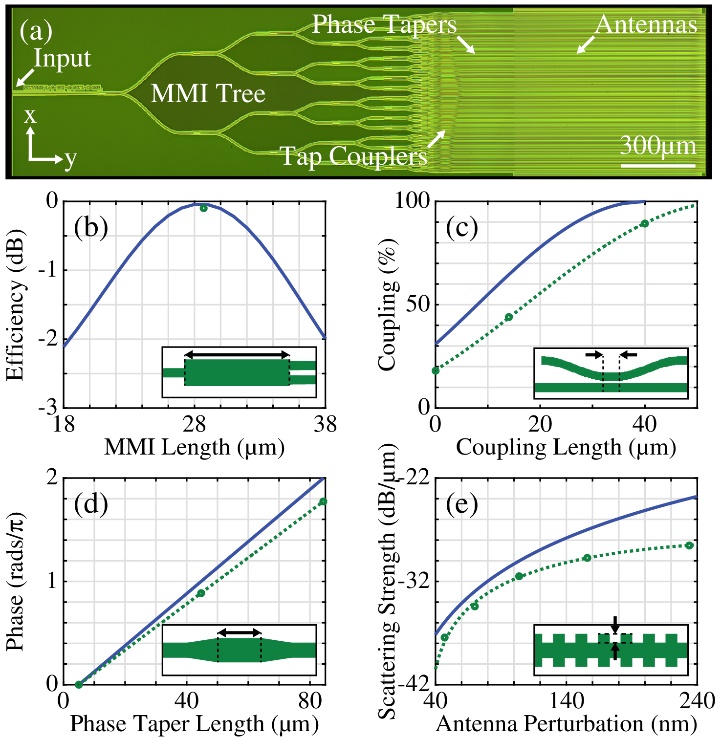 |
| 2018^3^ | < 100 mm  (Fig. 4) | -- | 1064 nm | -- | 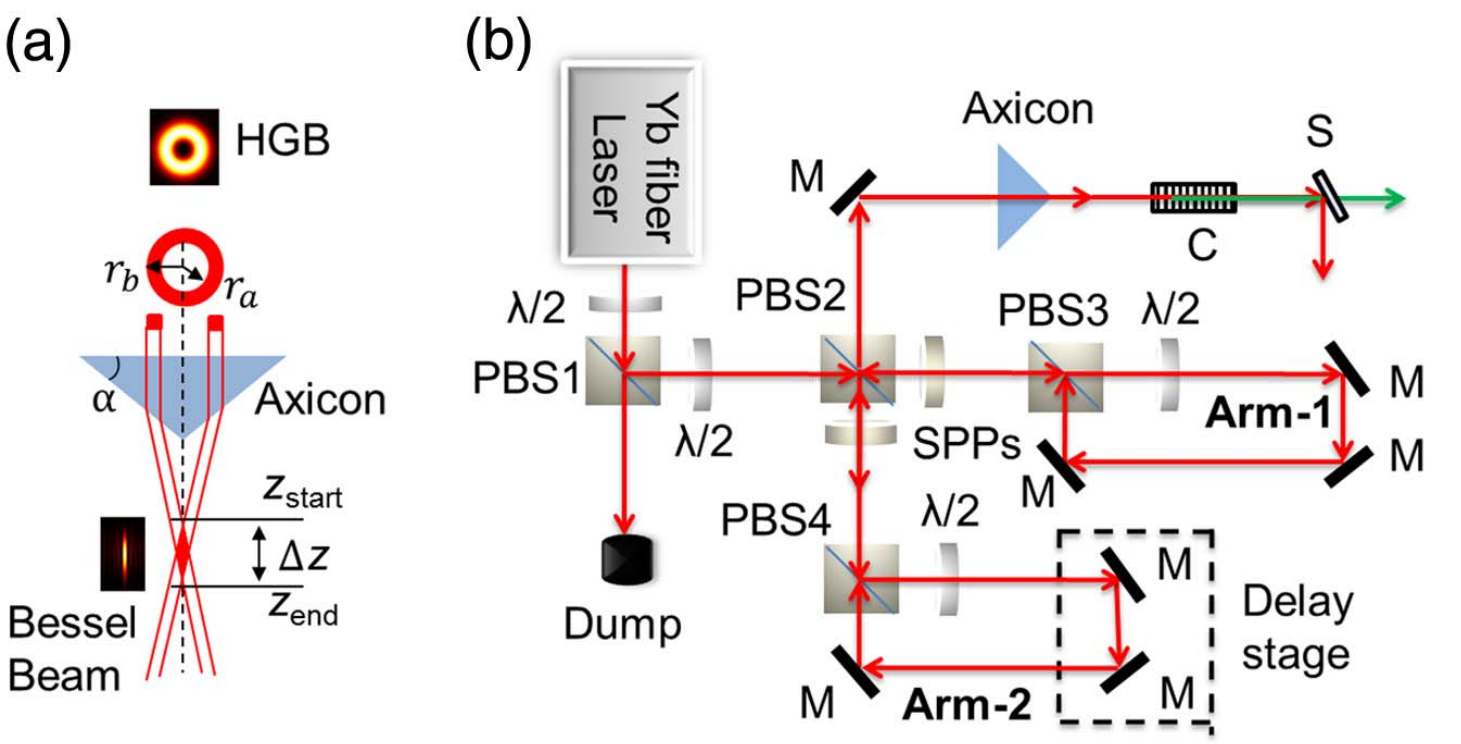 |
| 2020^4^ | 25 μm  (Fig. 5b) | Linear  polarization | 1.5-1.64 μm | 8.02 μm × 2.85 μm | 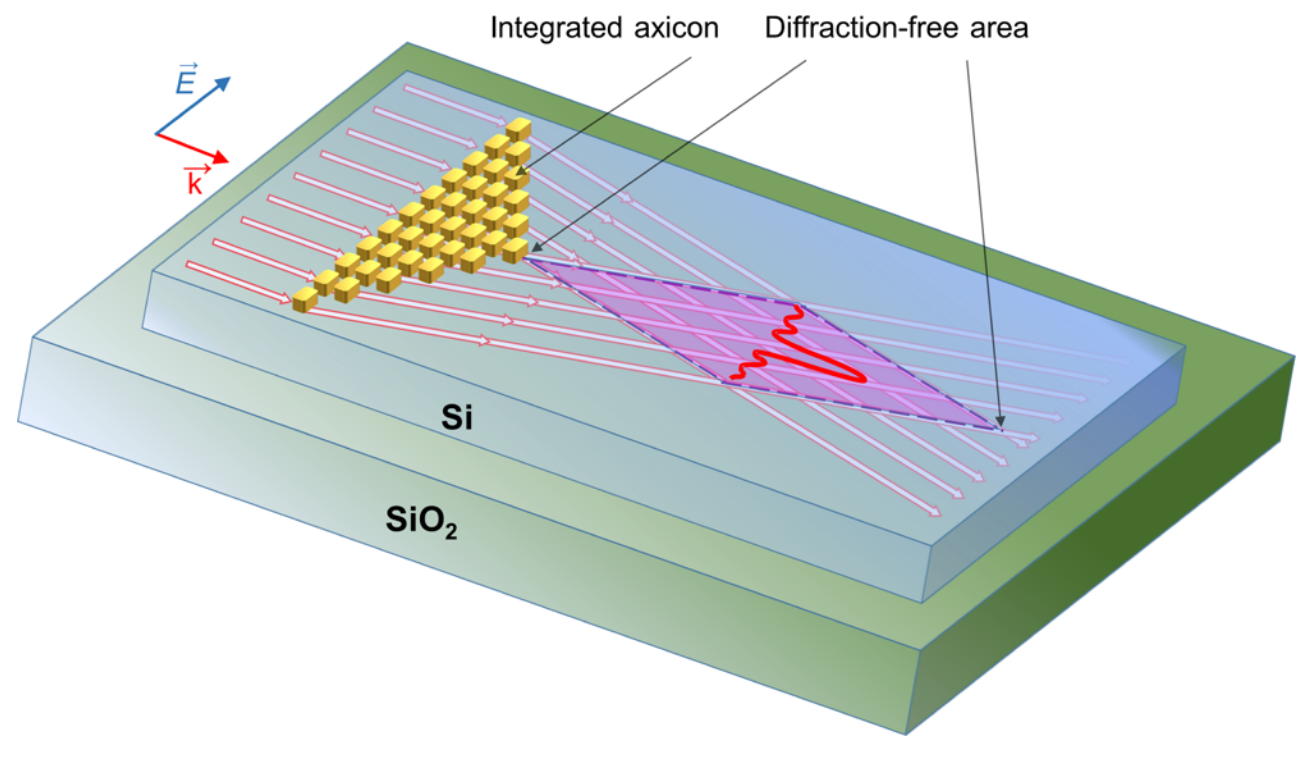 |
| 2021^5^ | ~130 mm | -- | 15 mm | 140 mm × 140 mm  (Fig. 3a) | 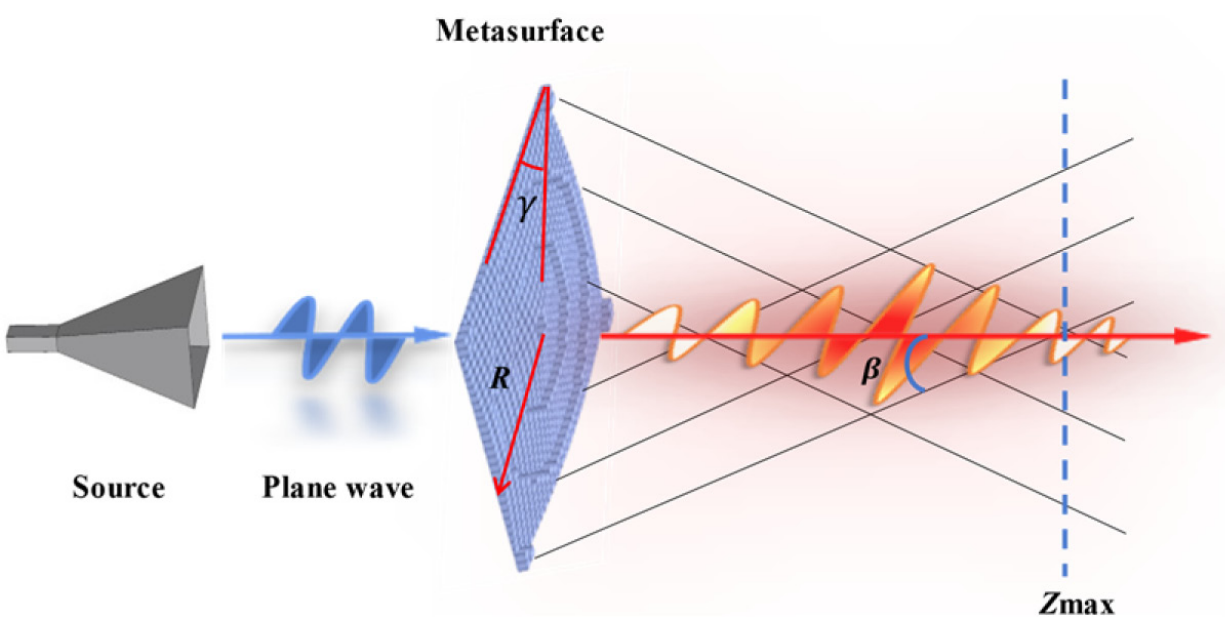 |
| 2022^6^ | < 200 mm  (Fig. 4) | -- | 3 mm | 101.6 mm | 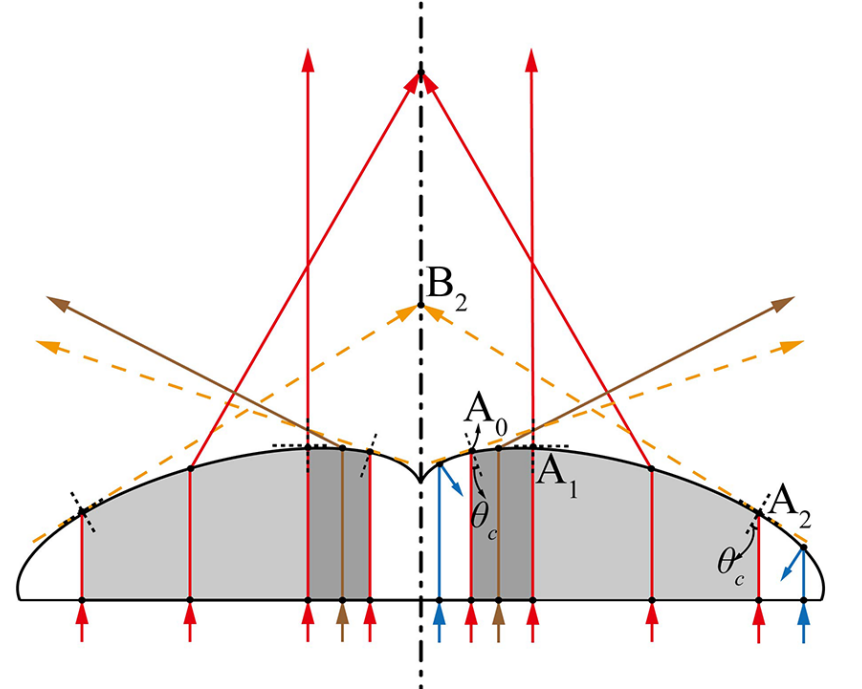 |
| 2022^7^ | ~230 μm | -- | 940 nm and 1064 nm | < 80 μm | 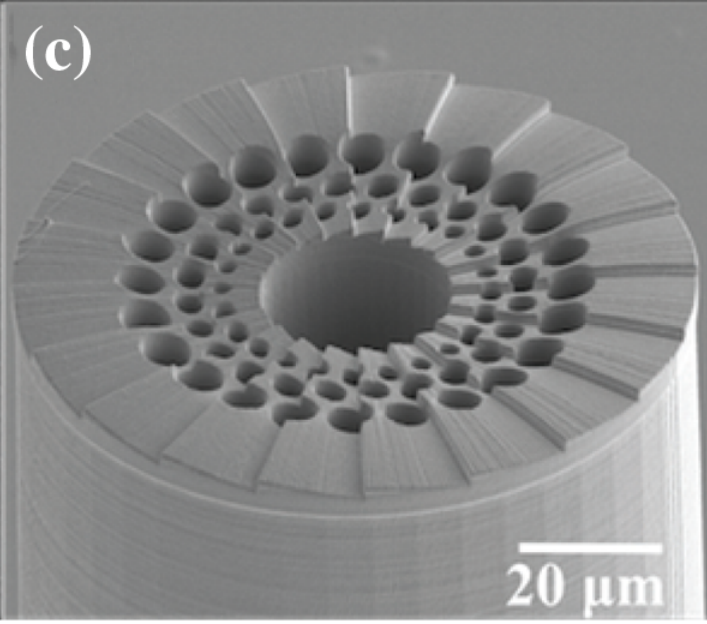 |
| 2022^8^ | 30 mm  (Fig. 2d) | -- | 2.14 mm | 40 mm  (Diameter) | 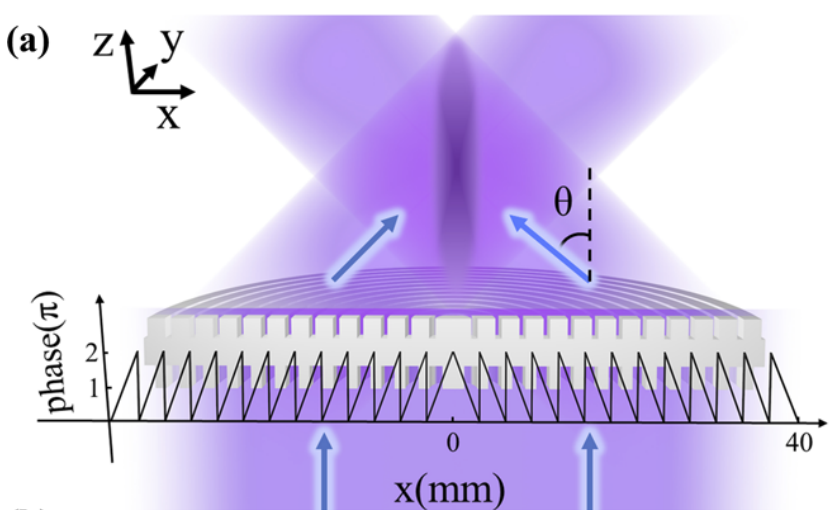 |
| This Work | Infinite  (theoretically)  10.24 m  (Measured) | azimuthally polarized | 1.5-1.63 μm | 0.87 mm  (Diameter) | 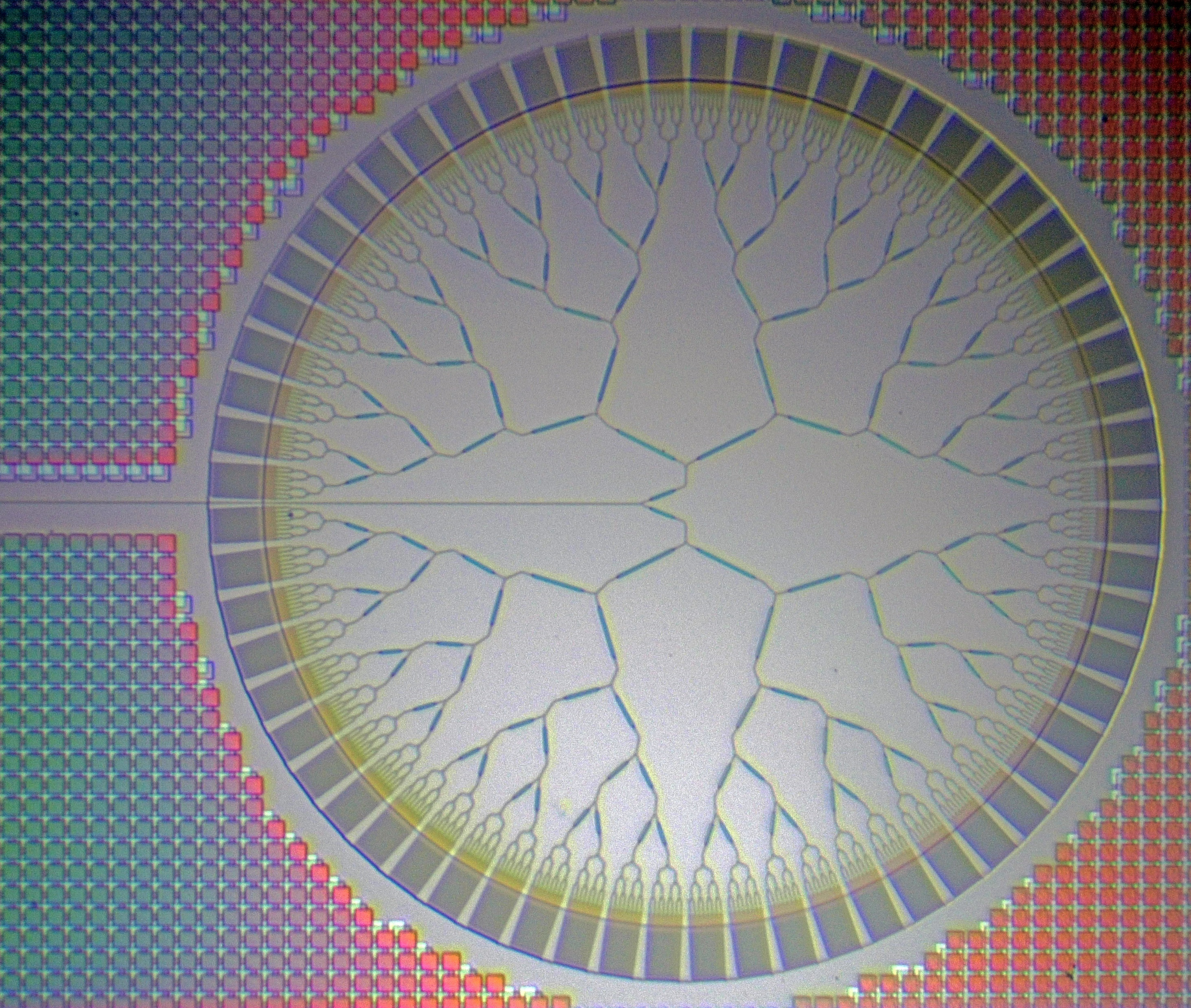 |

Section 2. Difference between Bessel beam and Bessel-Gaussian beam (BGb)

**
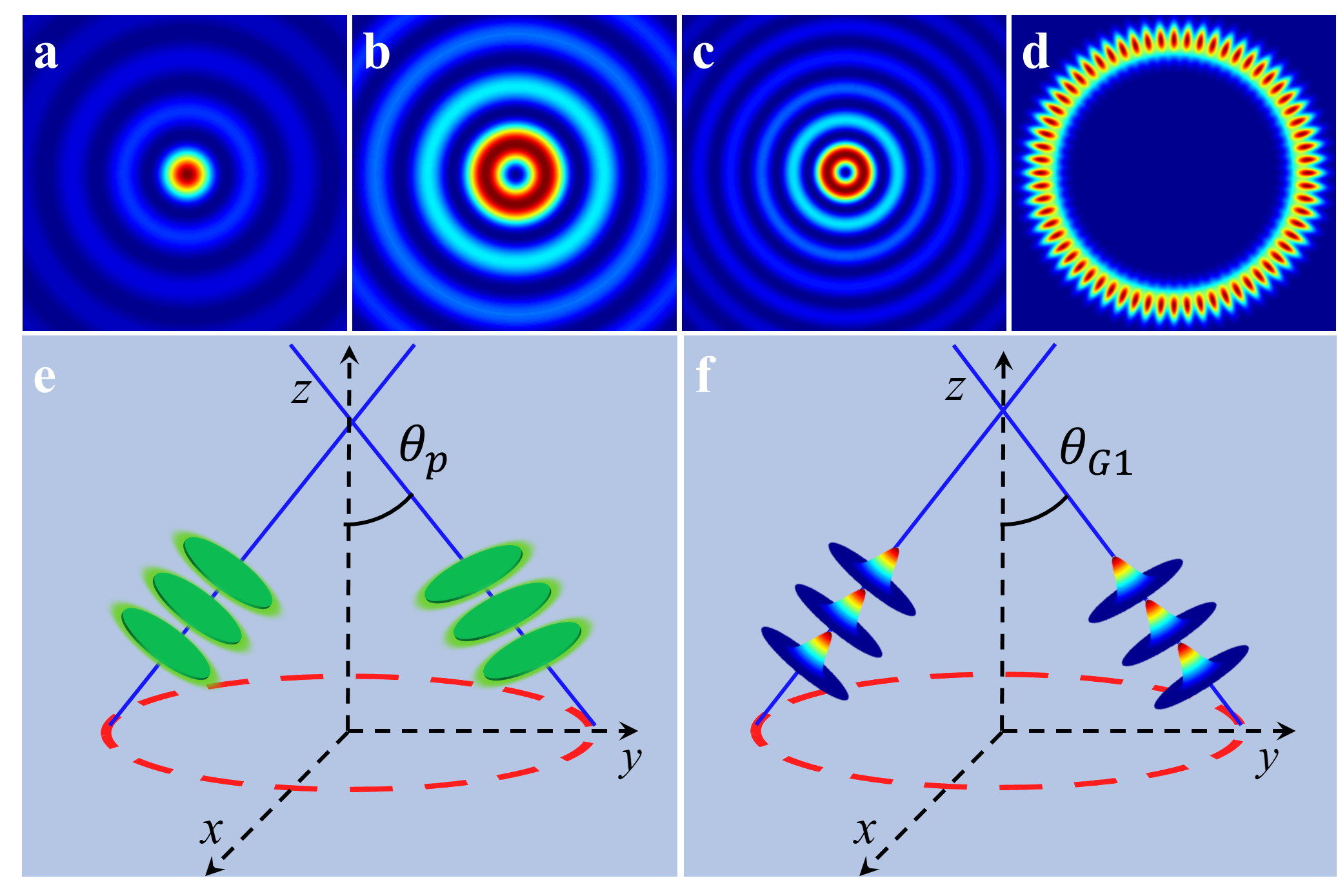
**

**Fig. S1. Bessel beam and Bessel Gaussian beam**. **a** Beam profile for an infinitely-expanded zero-order Bessel beam constructed by coherent super-imposition of plane waves at the same polarization states and initial phases. Each plane wave is emitted from the circularly distributed wavelet sources. **b** Interference superposition of Bessel beams by infinitely expanding the plane waves in the same initial phases, but the polarization of each individual wavelets is tangential to the circular distribution of wavelet sources. **c** Superimposed BGb by finite size Gaussian beams with a certain cone half angle, those wavelets have the same initial phase and different polarization. **d** All Gaussian beams do not overlap each other. The phase and polarization are the same as the former, except that cone half angle is large. **a-d** are simulation results from Equation (S2.1) and they have the same simulation height (See Section 3 for the principle and parameters of simulation). **e–f** Schematic illustration for the generation process of the Bessel beam and BGb respectively.

The superposition process of the Bessel beam and BGb is shown in Fig. S1e and S1f, which can be described by Equation (1)^9^.

（S2.1）

Where *Ψ_v_(ϕ_G_)* represents a series of wavelets that propagate at the angle *ϕ_G_* along circular cone towards its apex. The cone half angle is *θ*_P_ for plane wave and *θ*_G1_ for Gaussian wave. For plane waves, Bessel beam will be generated on the wavelet when 0 < *θ*_P_ < *π*/2. Since the ideal plane wave does not change on propagation and extends infinitely, the Bessel beam, formed by superposition, is also infinitely extended. Fig. S1a and Fig. S1b are the zero-order and first-order Bessel beams calculated by the above method, and their profiles do not change with the propagation distance.

For Gaussian beams, the above interference process is not only related to *θ*_G1_, but also to *θ*_G2_ = *λM*^2^/*πw*_0_, the divergence half-angle of Gaussian beams, where *λ* is the wavelength and *w*_0_ is the girdle radius of Gaussian, *M* is the beam quality factor. Fig. S1c shows the light intensity profile when these Gaussian beams overlap and Fig. S1d is before the overlap (such as the emitter surface) or after the overlap. The Gaussian wavelet have no overlap since the larger *θ*_G1_ in Fig. S1d.

In optical phased arrays, the cone half angle is the emission angle *θ*_G1_ = *arcsin*(*n_eff_* - *λ*_0_/Λ), where Λ is the grating period, *n_eff_* is the effective refractive index of the grating, and *λ*_0_ is the wavelength. When the device is fixed, it decreases as the wavelength increases. With the knowledge of geometrical optics, there are the following 4 cases in Table S2. The schematic diagram of each case is shown in Fig. S2.

**
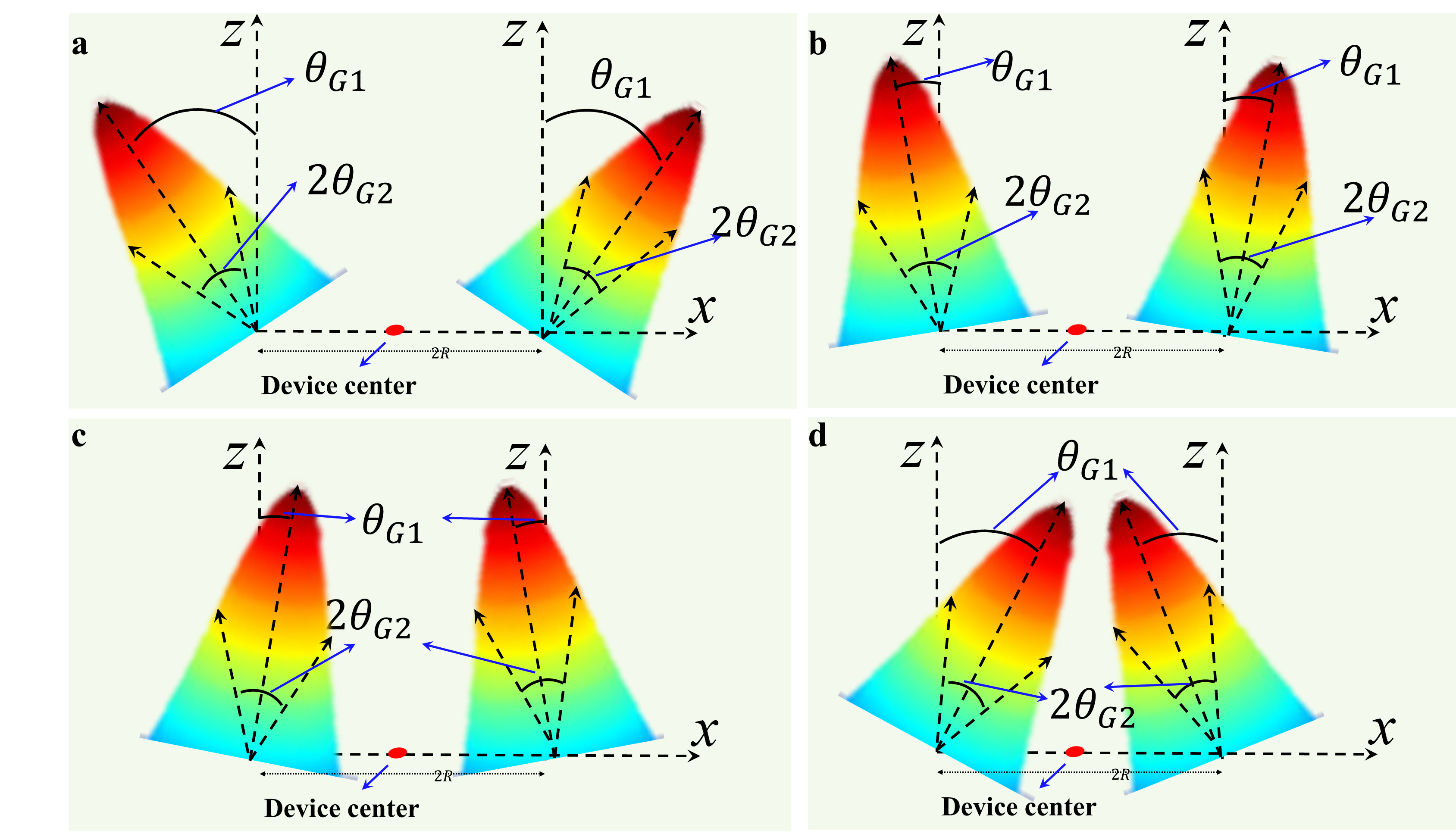
**

**Fig. S2. Schematic diagram for the generation of Bessel Gaussian beam. a** BGb cannot be generated due to separated Gaussian beams. **b**, and **c** infinite BGb can be generated due to overlapping Gaussian beams. **d** The generated BGb only exists within a limited distance due to separation of Gaussian beams after overlapping.

**Table S2.** **The Relationship between the propagation distance of BGb Generated by Integrated Photonic Chip with emission angle *θ_G_*_1_ and divergent half-angle *θ_G_*_2_.**

| **Case** | *θ*_G1_ | |*θ*_G1_*|-θ*_G2_ | *Z_start_* | *Z_end_* |
| --- | --- | --- | --- | --- |
| **1** | ≥ 0 | > 0 | non-existent | non-existent |
| **2** | ≥ 0 | ≤ 0 | *R*/*tan*(*θ*_G2_ - *θ*_G1_) | +∞ |
| **3** | < 0 | ≤ 0 | *R*/*tan*(*θ*_G2_ - *θ*_G1_) | +∞ |
| **4** | <0 | > 0 | *R*/*tan*(*θ*_G2_ - *θ*_G1_) | *R*/*tan*(*-θ*_G2_ - *θ*_G1_) |

When *θ*_G2_ ≥ |*θ*_G1_*|*, all Gaussian beams always overlap and interfere in the *z* direction, which means that BGb always exists beyond *Z_start_* = *R*/*tan*(*θ*_G2_ - *θ*_G1_), where *R* is the radius of the device. Due to the divergence of the Gaussian beam, the BGb spot becomes larger as the propagation distance increased in this case. When *θ*_G2_ < |*θ*_G1_*|* all Gaussian beams will only overlap within a certain distance as shown in Case 4 and Fig. S2d. Beyond the propagation distance of *Z_end_* = *R*/*tan*(*-* *θ*_G2_ - *θ*_G1_), the Gaussian beams spread separately again.

Section 3. Simulation details

**Simulation detail 1: The simulation process for generating the Bessel beams shown in Fig. S1a and Fig. S1b**


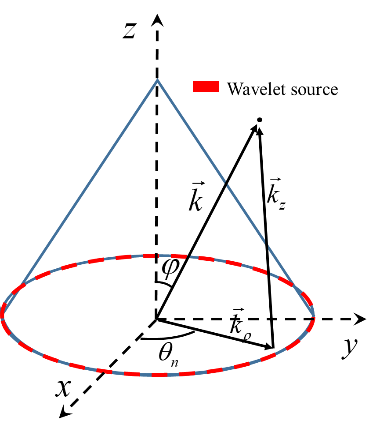


**Fig. S3. Schematic illustration for the generation process of the Bessel beam and the BGb respectively.**

The scalar and vector electric field superimposed by equal phase planes from the 64 wavelet sources can be expressed respectively by

(S3.1)

(S3.2)

Where *a_n_* is the amplitude of the *n-th* plane wave, *θ_n_* = 2*πn*/64 is the azimuthal angles, $\vec{k}_{n}$ = *k*(*sinφcosθ_n_*, *sinφsinθ_n_*, *cosφ*) is the wave vector for the *n-th* field, *φ* = *arcsin*(*k_ρ_*/*k*) is a polar angle indicating half-angle of the cone illustrated in Fig. S3^14^, *k* = *2π/λ* is the wavenumber, *λ* is the wavelength of plane beam in vacuum and *l* is the topological charge. Noted that the wavelet sources (also known as “beam-emitted grating”) are allocated at the plane of (*x_n_*, *y_n_*, 0) and the image is capture at the plane of (*x*, *y*, *z*). Based on Eq. (S3.1), the Fig. S1a can be obtained by using the following parameters: *x* and *y* are from -6 μm to 6 μm , *z* = 0.02 m, *a_n_* = 1, *λ* = 1.55 μm, *l* = 0, and the polar angle is *φ* = 25°. To obtain Fig. S1b, the abovementioned parameters are still substituted into Eq. (S3.2). In particular, since the polarization state of each individual wavelet sources changes with the angular angle *θ_n_* , the term of (*sinθ_n_*, *- cosθ_n_*)*^T^* in Eq. (S3.2) are substituted by *θ_n_* = 2*πn*/64 accordingly.

**Simulation detail 2: The generation of Bessel Gaussian beam shown in Fig. S1c-d and Fig. 2a-d**

The beam profile for Bessel Gaussian beam is finite due to limited beam waist of the Gaussian beam. Hence, the electric field of the upward-emitted Gaussian beam can be described by

$E_{00}\left(x,y,z\right)=a_{n}\frac{w_{0}}{w\left(z\right)}H_{0}\left(\frac{\sqrt{2}x}{w\left(z\right)}\right)H_{0}\left(\frac{\sqrt{2}y}{w\left(z\right)}\right)\exp\left(-\frac{x^{2}+y^{2}}{w^{2}\left(z\right)}\right)\exp\left[i\Psi\left(x,y,z\right)\right]$ (S3.3)

Where *a_n_* is the amplitude,$w\left(z\right)=w_{0}\sqrt{1+\left(\lambda/\pi w_{0}^{2}\right)^{2}}$, *w*_0_ is the beam waist, *H*_0_(x) represents the Hermite polynomials, *Ψ*(*x*, *y*, *z*) is the phase of the electric field.

In order to superimpose the electric fields of Gaussian beam, the spatial rotation of the fields is required, as shown in Fig. S1f. The coordinate for the image, the wavelet source and the Gaussian electric field after rotation are (*x*, *y*, *z*), (*x_n_*, *y_n_*, *z_n_*) and (*x*″, *y*″, *z*″), respectively. *θ_n_* = 2*πn*/64 is the azimuthal angles, and *φ* is a polar angle indicating half-angle of the cone. The relation can be expressed by

(S3.4)

The center position of each beam is (*x_n_*, *y_n_*, *z_n_*) = (*R*cos*θ_n_*, *R*sin*θ_n_*, 0) and the corresponding polarization direction is (*sinθ_n_*, -*cosθ_n_*, 0)*^T^*, where *R* is the radius of the device. The BGb can be obtained by

$\vec{E}_{BGb}=\sum_{n=1}^{n=64} a_{n}\left(\begin{matrix} \sin\theta_{n} \\ -\cos\theta_{n} \\ 0 \end{matrix}\right)\frac{w_{0}}{w\left(z''\right)}H_{0}\left(\frac{\sqrt{2}x''}{w\left(z''\right)}\right)H_{0}\left(\frac{\sqrt{2}y''}{w\left(z''\right)}\right)\exp\left(-\frac{x''^{2}+y''^{2}}{w^{2}\left(z''\right)}\right)\exp\left[i\psi\left(x'',y'',z''\right)\right]$ (S3.5)

Where *Ψ*( *x*″, *y*″, *z*″) = *k*(*x*″*^2^* +*y*″*^2^*)/(2*R*(*z*″))+*kz*″–*arctan*(*z*″*/f*), and *R*(*z*″) = *z*″*+f* ^2^*/z*″*. f* = *πw*_0_^2^/*λ* is the Rayleigh length. The simulation parameters are as followed, *z* = 0.02 m, *a_n_* = 1, *λ* = 1.55 μm, *R* = 0.43 mm, *w*_0_ = 0.02 mm. The Fig. S1c shows the simulated BGb profile in the case of *x* and *y* are from -0.2 mm to 0.2 mm, and *φ* = 1°. Fig. S1d shows the simulated BGb profile in the case of *x* and *y* are from -4 mm to 4 mm, and *φ* = 10°.

Based on Eq. S3.5, the components of the BGb electric field *E_x_* and *E_y_* can be extracted. According to the polarization characteristics of the space beam, the BGb profiles in different polarization states can be obtained, as shown in Fig 2 b-e in the revised manuscript. The simulation parameters for the BGb profile in **Fig. 2a-e** in the revised manuscript are as followed: ***z* = 2.2 m**, *a_n_* = 1, *λ* = 1.55 μm, *R* = 0.43 mm, *w*_0_ = 0.02 mm. *x* and *y* are from -2 cm to 2 cm, and *φ* = 1°.

**Simulation detail 3: The generation of Bessel Gaussian beam emitted from grating arrays shown in Fig. 1c**

For two-dimensional grating arrays, the coordinate for the far-field BGb is shown in Fig. S4a. The center of the grating arrays is located at the coordinate origin.


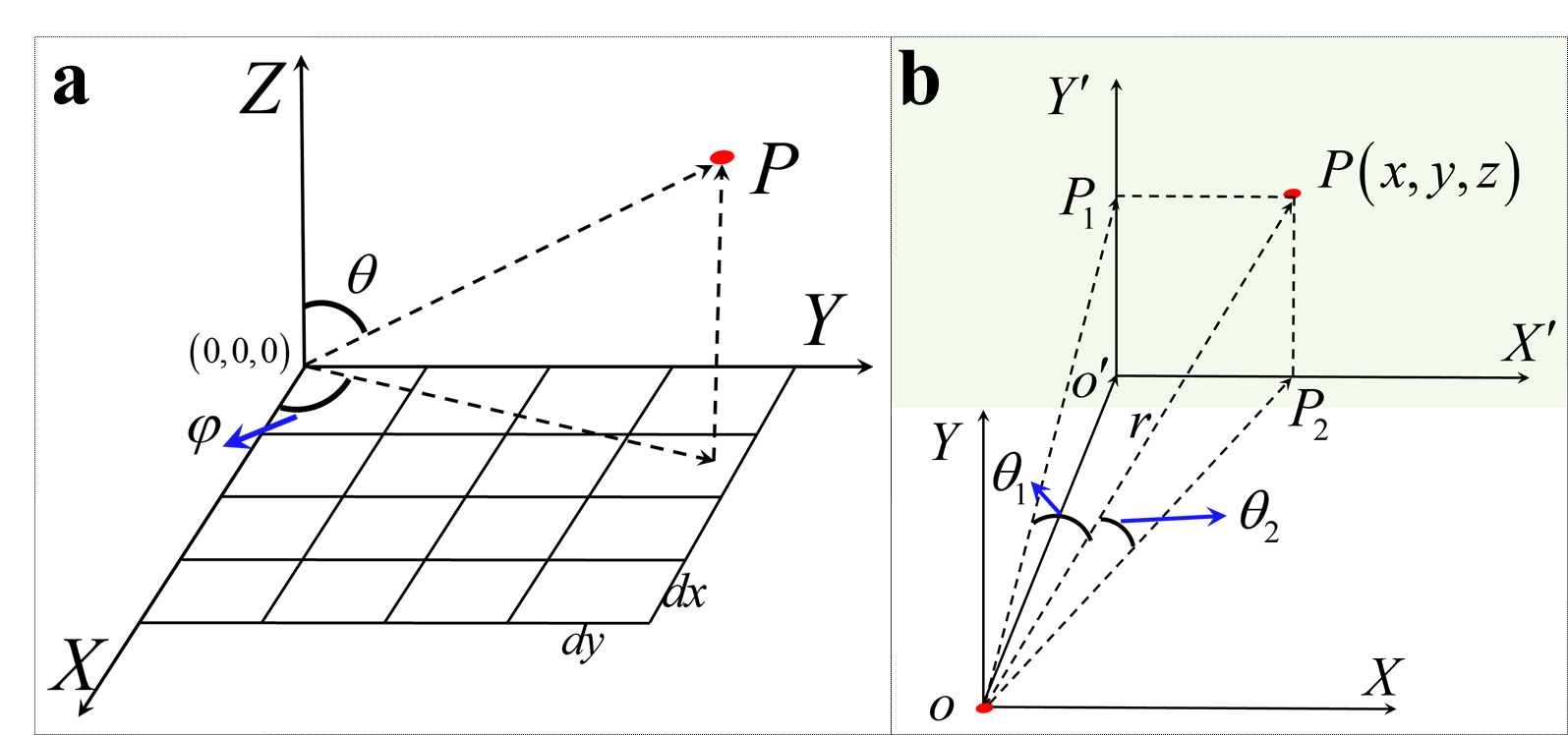


**Fig. S4a Schematic coordinate for the far-field BGb generated by grating arrays. b** **Schematic diagram of transverse angle** *θ*_1_ **and longitudinal angle** *θ*_2_ **of the beam emitted by grating array.**

The electric field of the BGb can be expressed by

(S3.6)

Where *m* is the serial number of the waveguide, *n* is the serial number of the grating period, *a_mn_* is the amplitude, *j* is the imaginary number, *d_x_* is the grating period, *d_y_* is the waveguide spacing, and *φ_mn_* is the initial phase related to the transmission distance. The parameters for the grating array are as followed, *d_x_* = 800 nm, *d_y_* = 1.62 μm. Considering the transmission loss in the waveguide, the amplitude of each cycle is exponentially attenuated. The attenuation can be defined as *a_mn_* = *A_m_*_1_*α*(*^n^*^-1)^ and *A_m_*_1_ = 1 along the *x* direction, and *α* is attenuation coefficient. Finally, the Fig. 1c shown in the revised manuscript can be obtained by applying *α* = 0.85, *z* = 0.1 m to the simulation. The angle of the beam from the grating array along the *x* and *y* directions is *θ*_1_ and *θ*_2_ respectively, as shown in Fig S4b. The distance from the far field point (*x*, *y*, *z*) to the center of the grating array is *r*, then *r*^2^ = *x*^2^ + *y*^2^ + *z*^2^, and *x* = *r*sin*θ*_1_, *y* = *r*sin*θ*_2_. We set *θ*_1_ from -15 ° to 15 ° and *θ*_2_ from -10 °to 10 ° in the Fig. 1c.

Section 4. Design of waveguide structure

The Fig. S2 and Table S2 indicate that the generation of infinite BGb requires careful design of waveguide structure, especially grating arrays width and grating period, to meet the relationship of *θ_G2_* ≥ |*θ_G1_|*. First of all, we calculate the divergence half-angle *θ_G2_* under different attenuation coefficients *α* according to the two-dimensional grating array model (Eq. S3.6). As shown in Fig. S5a, the normalized intensity decays exponentially along the waveguide direction. The smaller the attenuation coefficient, the faster the normalized intensity decays. Fig. S5b shows the far-field spot profile when the attenuation coefficient *α* is 0.95, and the one-dimensional section along the waveguide transmission direction. The distribution of electric field in the Fig. S5b is a two-dimensional Gaussian profile, in which the divergence angles of the two directions are different. The angle corresponded to 1*/e*^2^ of peak power is defined as the divergence angle. Hence, the calculated divergence angle along the waveguide direction is 3.26 °. As shown in Fig. S5b to Fig. S5f, the divergence angle evolves from 3.26 ° to 14.95 ° as the attenuation coefficient *α* varied from 0.95 to 0.8.

We calculate the divergence half-angle using the data that the normalized power drops to 1*/e*^2^ of the peak value. However, it is worth mentioning that the definition is too standardized. Since the photonic chip has 64 channel grating arrays, even though the light intensity of each array attenuates to 5% of the peak, after 64 channels are stacked, the light power directly above the chip center is still higher than the peak power of a single grating array. So that it can be seen through the infrared camera with high exposure time. This shows that the divergence angle of the light spot observed in the experiment is larger than the divergence angle calculated under this definition. However, since there is no better-known definition, we still need to use this definition to make some quantitative explanations. The simulation parameters are as follows: *d*_x_ = 800 nm, *d*_y_ = 1.62 μm, *z* = 0.1 m, far-field *x*_0_ is from -2 cm to 2 cm, and *y*_0_ is from -3 cm to 3 cm.


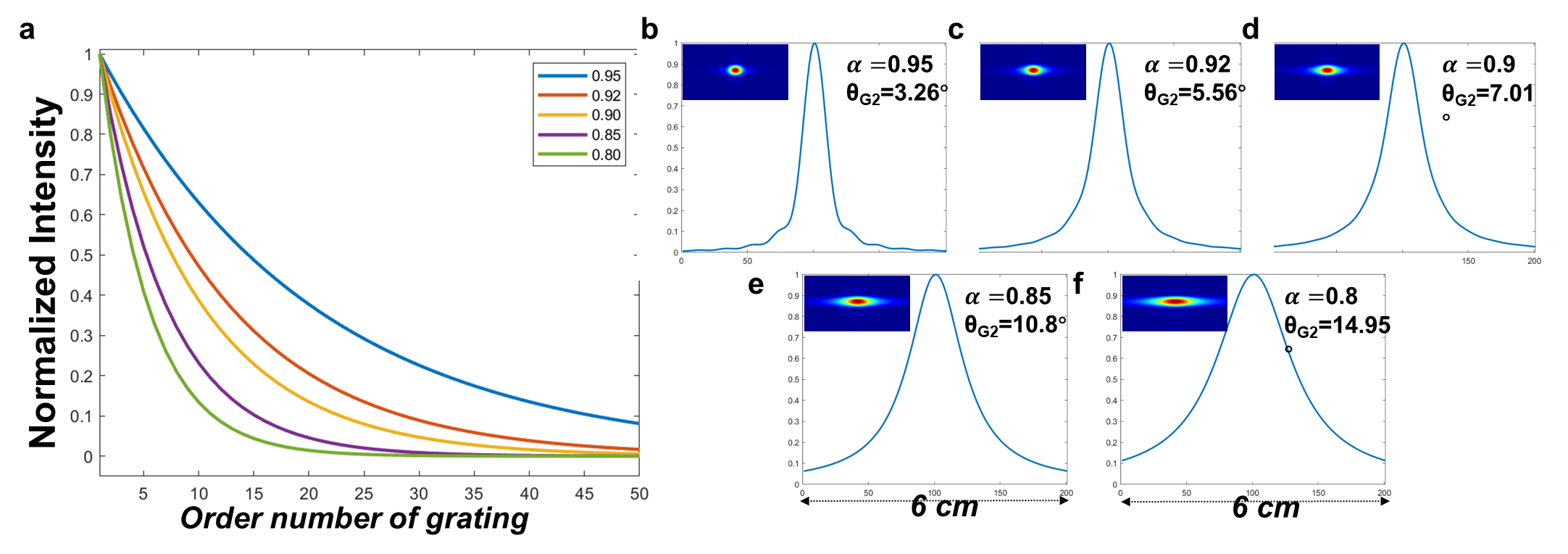


**Fig. S5.** **The divergence half-angle *θ_G_*_2_ under different attenuation coefficients. a** The intensity of light emitted from the grating array decreases exponentially with the increase of grating period. Grating period is 0.8 μm. The grating array 50 cycles for 40 μm long. Far field intensity profile and divergent half-angle under different attenuation coefficients: **b** *α* *=* 0.95, **c** *α* *=* 0.92, **d** *α* *=* 0.9, **e** *α* *=* 0.85, **f** *α* *=* 0.8.

In fact, it is not difficult to calculate that the angle does not change much with the waveguide width. The following simulation results are available. (a), 400 nm × 800 nm: 3.26 °, 5.48 °, 6.84 °, 10.70 °, 14.95 °; (b), 380 nm × 800 nm: 3.26 °, 5.56 °, 7.01 °, **10.80 °**, 14.95 ° corresponding attenuation coefficients are 0.95, 0.92, 0.90, 0.85, 0.80 respectively. The simulation results here show that the waveguide width has little effect on the divergent half angle.

Next, we use Lumerical Finite Difference Time Domain (FDTD) tools to simulate the influence of the width and period of the grating array on the emission angle *θ_G1_*. As shown in Fig. S6a, the emission angle of the grating decreases with the increasing incident wavelength. We scanned several groups of different grating periods in Fig. S6a, waveguide widths in Fig. S6b, and thus obtained the range of grating emission angles correspondingly. The purpose is to find the structure sizes that meet the condition of *θ_G2_* ≥ |*θ_G1_|*.


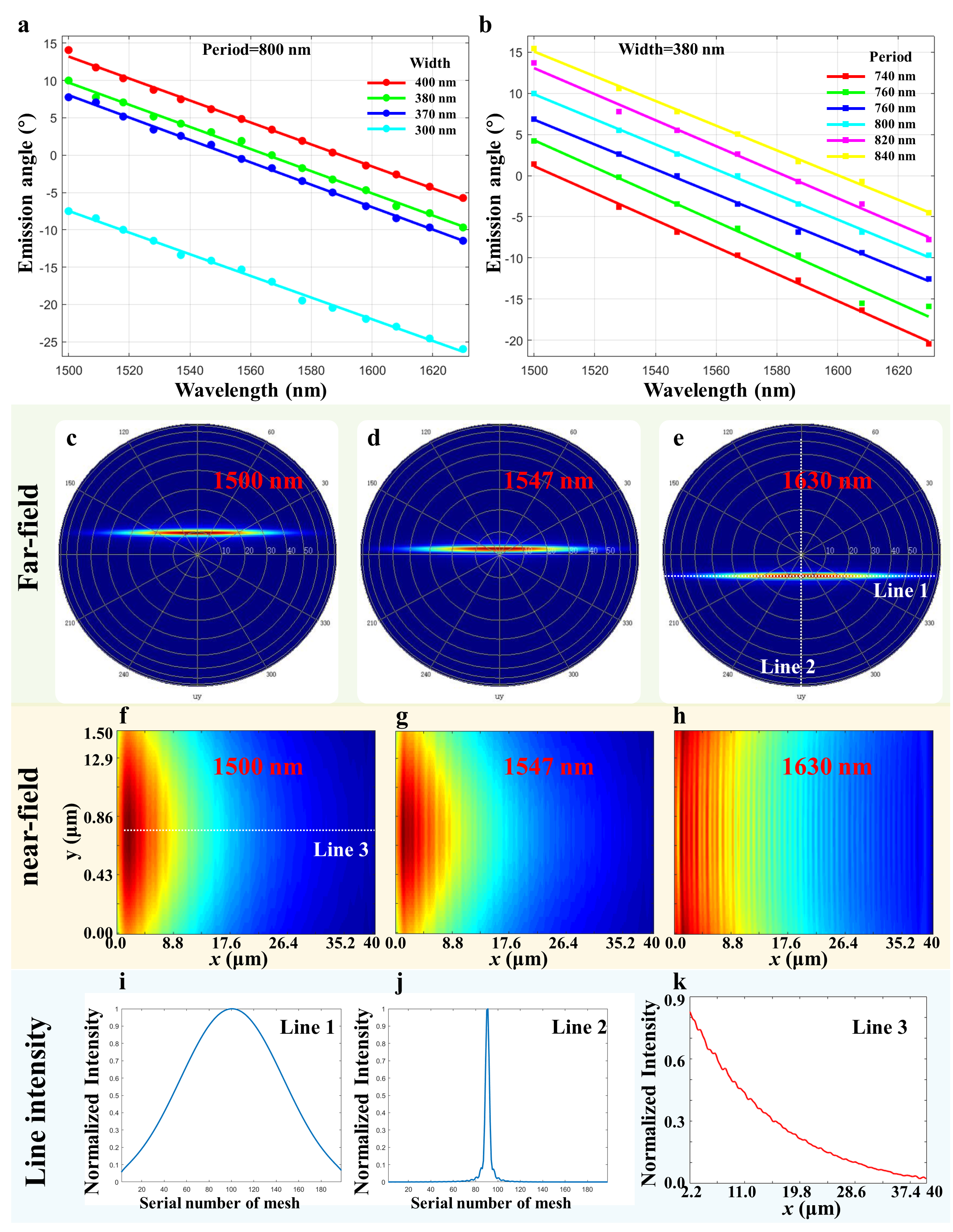


**Fig. S6.** **The Emission angle *θ_G1_* is tuned with the operating wavelength and waveguide structure.** **a** The plot for the emission angle against the operating wavelength with waveguide widths being varied from 300 nm to 400 nm and the grating period being fixed at 800 nm. **b** The plot for the emission angle against the operating wavelength under the grating period varied from 740 nm to 840 nm and the waveguide width being fixed at 380 nm. The scattered points indicate the simulation data and the solid line are the first-order polynomial fitting. **c-e**, Beam profiles at the far field when the operating wavelengths are changed with 1500 nm, 1547 nm, and 1630 nm respectively. **f-h**, Beam profiles at the near field when the operating wavelengths are tuned at 1500 nm, 1547 nm, and 1630 nm respectively. **i** and **j** are the cross sections of the far-field beam profile operated at 1630 nm along the Line 1 and Line 2 directions shown in **e**, respectively. **k** is the normalized intensity profile truncated along the Line 3 direction shown in **f**. It is located at the center of the near-field beam profile operated at 1500 nm.

Furthermore, we calculate the far-field (shown in Fig. S6c-e) and near-field (shown in Fig. S6f-h) beam profiles of the grating array at different operating wavelengths. Fig. S6i and Fig. S6j are the cross sections of the far-field beam profile operated at 1630 nm along the Line 1 and Line 2 direction indicated by white dash lines in Fig. S6e, respectively. Both of them indicate that a Gaussian-like beam can be constructed in the far field via the beams emitted from the grating array Fig. S6k is the normalized intensity profile truncated along the Line 3 direction indicated by a white dash line in Fig. S6f. The line is located at the center of the near-field beam profile operated at 1500 nm. The exponentially decayed distribution is mainly due to the transmission loss in the waveguide. The non-smooth phenomenon in the image comes from the mesh setting in the FDTD. The parameters used in Fig. S6c-k are as followed, waveguide: 380 nm × 220 nm, grating period: 800 nm, number of periods: 50, duty ratio: 0.5, etching depth: 70 nm, wavelength: 1500 nm-1630 nm. The monitor with a size of 1.5 μm × 40 μm is located at 1 μm directly above the grating array and a cell mesh size is 22 nm × 22 nm.

The simulation results show that for a grating array with a waveguide width of 400 nm and a grating period of 800 nm, its emission angles at the incident wavelengths of 1500 nm and 1630 nm are 14.08 ° and -5.76 ° respectively. For the convenience of expression, we record it as (400 nm, 800 nm, 14.08 °, -5.76 °). The simulation results are as follows: (400 nm, 800 nm, 14.08 °, -5.76 °), **(380 nm, 800 nm,** **10.0 °, -9.69 °)**, (370 nm, 800 nm, 7.76 °, -11.47 °), (300 nm, 800 nm, -7.5 °, -25.98 °). The results of scanning grating period with fixed waveguide width are as follows: (380 nm, 740 nm, 1.39 °, -20.46 °), (380 nm, 760 nm, 4.23 °, -15.90 °), (380 nm, 780 nm, 6.84 °, -12.58 °), (380 nm, 800 nm, 10.0 °, -9.69 °), (380 nm, 820 nm, 13.68 °, -7.76 °), (380 nm, 840 nm, 15.45 °, -4.50 °). Based on the above simulation results, we have found that a 380 nm width grating array at a period of 800 nm can support infinite BGb over the wavelength range from 1500 nm to 1630 nm.

The BGb generated by the photonic chip can be measured at the infinitely far field as the wavelength changes from 1500 nm to 1630 nm, only the initial position of BGb change accordingly and the far-field profile still can be maintained. This is the reason that we can measure the intensity profile of BGb beyond 10 m within the wavelength range. The measurable distance of the BGb generated by the integrated photonic chip can be clearly shown in the Fig. S7. We use the Finite Difference Time Domain (Lumerical FDTD) tools to simulate the change of the emission angle of grating array against the incident wavelength. The simulated wavelength range is from 1500 nm to 1830 nm and the emission angle decreases from 10.0 ° to -42.4 °. Since the divergence half-angle *θ_G2_* is 10.8 ° (a waveguide width of 380 nm and a grating period of 800 nm, *α* = 0.85), the corresponding *Z_start_* (red lines) and *Z_end_* (blue lines) can be obtained via the formula in Table S2, respectively.


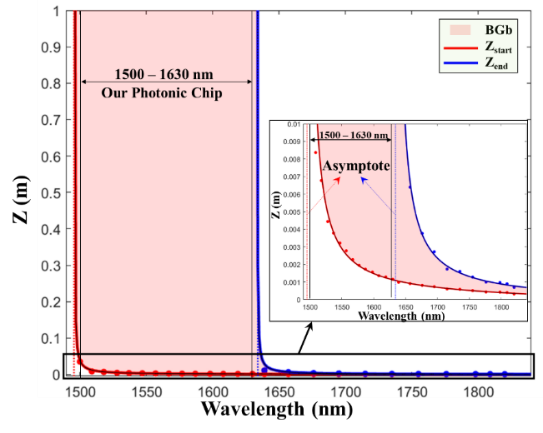


**Fig. S7. The measurable distance of BGb varies with the wavelength of incident light.**

As shown in the Fig. S7, the start position *Z_start_* (red lines) and the end position *Z_end_* (blue lines) of BGb can be asymptotic to 1495.2 nm and 1634.4 nm, respectively. The red shadow area between these two lines is the space where BGb can exist. Since the working range of our proposed photonic chips from 1500 nm to 1630 nm locates at the red shadow area, it indicates that the generated BGb can be transmitted to infinity. It can be predicted that the existence length of BGb could be limited but measurable, when the wavelength of the incident light increases beyond 1634.4 nm. For example, the simulation results show that when the incident light wavelength is 1695 nm, *Z_end_* = 2.72 mm, *Z_start_* = 0.73 mm, so the generated BGb is about 2 mm. However, this short distance BGb has been reported extensively in Table S1 in the supplementary.

Section 5. BGb profiles of different wavelengths are generated by integrated photonic chip at 1.55 m, 5.91 m, and 10.24 m.


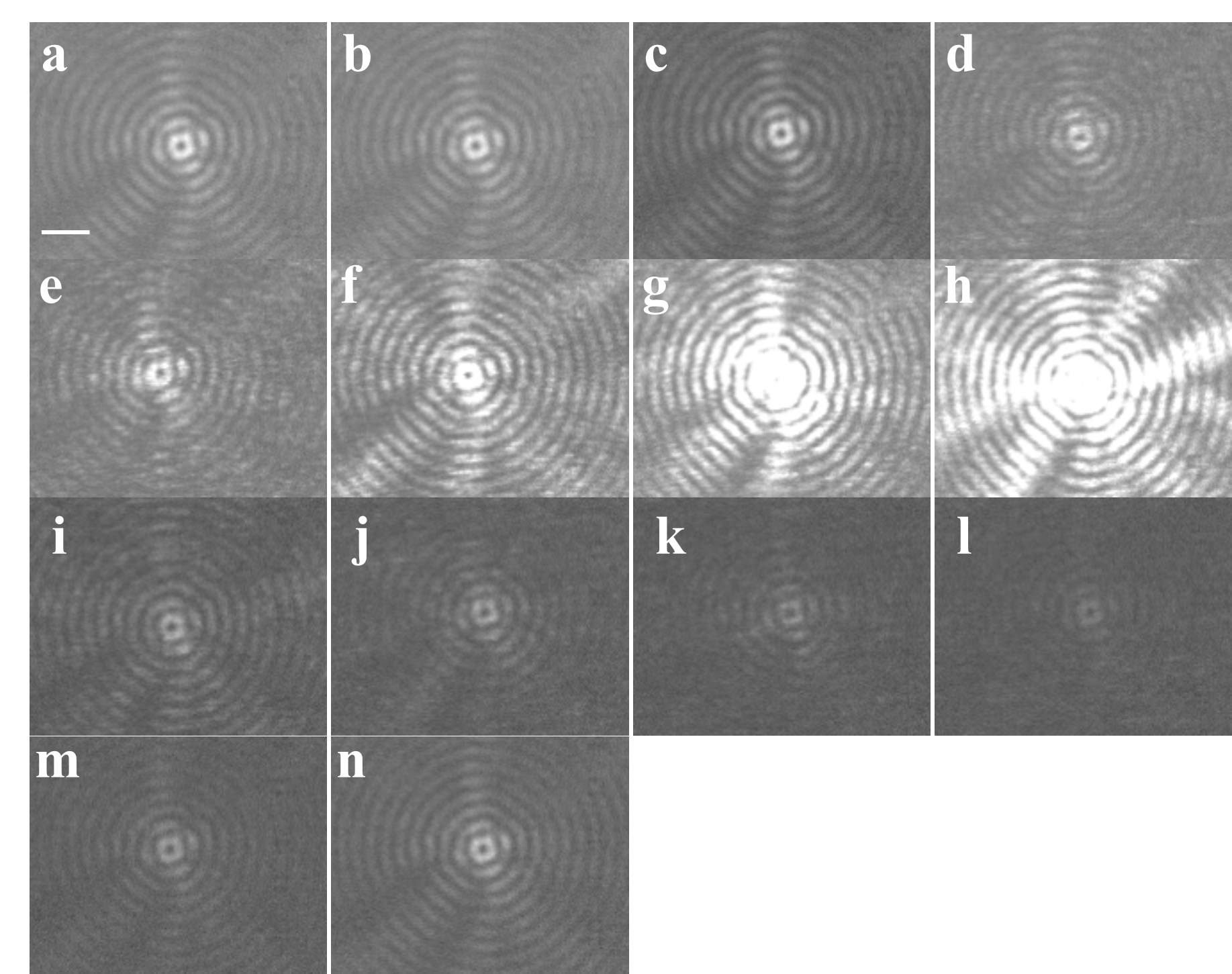


**Fig. S8. Experimentally measured *J*_1_ BGb profile for different wavelength at 1.55m.** **a** to **n** correspond to incident light with wavelengths of 1500 to 1630 nm, in steps of 10 nm, respectively. The scale bar is 1 cm and applies to all plots.


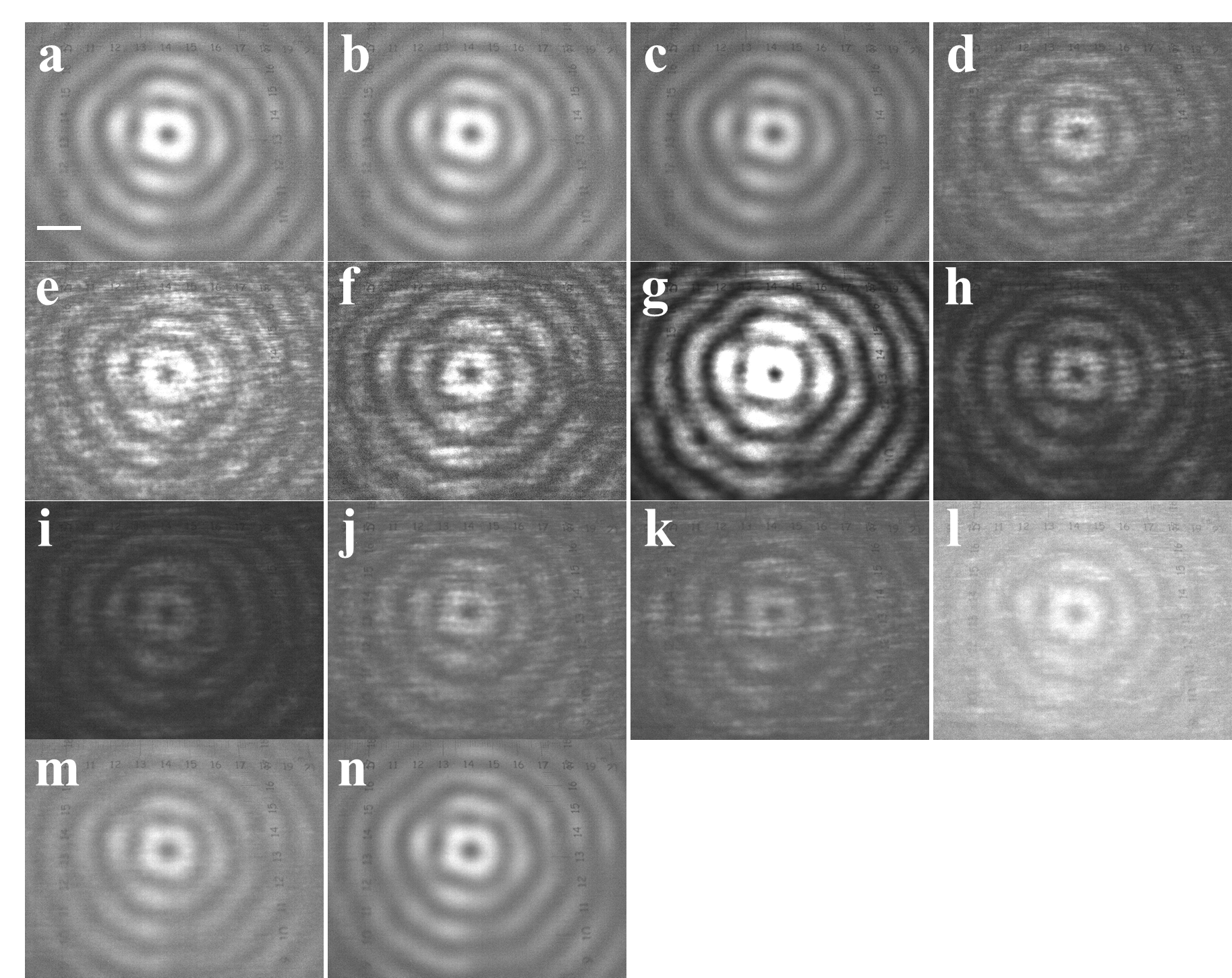


**Fig. S9. Experimentally measured *J_1_*BGb profile for different wavelength at 5.91m.** **a** to **n** correspond to incident light with wavelengths of 1500 to 1630 nm, in steps of 10 nm, respectively. The scale bar is 2 cm and applies to all plots.


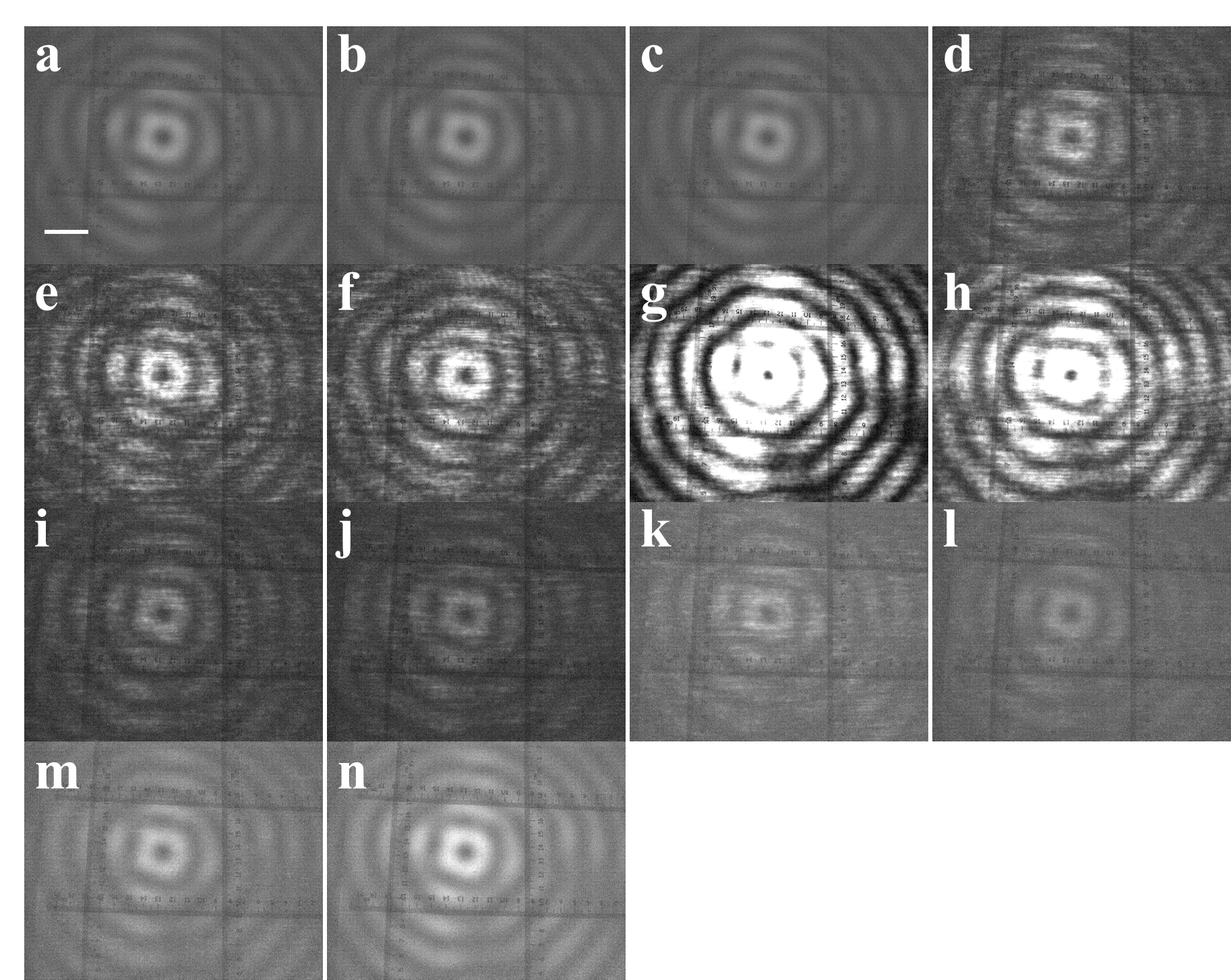


**Fig. S10. Experimentally measured *J_1_*BGb profile for different wavelength at 10.24 m.** **a** to **n** correspond to incident light with wavelengths of 1500 to 1630 nm, in steps of 10 nm, respectively. The scale bar is 3 cm and applies to all plots.

**
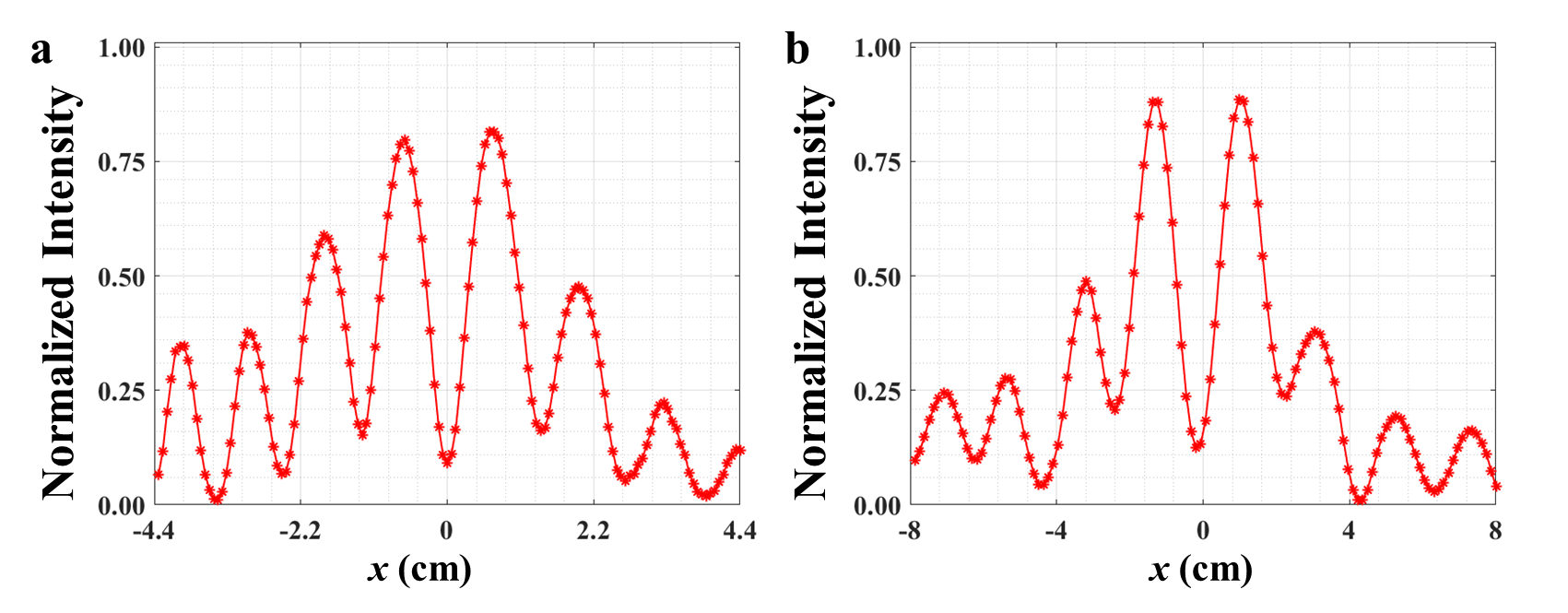
**

**Fig. S11. Normalized vertical section of BGb profile following *J*_1_ order of first-kind of Bessel function.** **a** The One-dimensional intensity distribution of the light field measured at 5.91 m emitted from a 1630-nm laser. **b** The One-dimensional intensity distribution of the light field measured at 10.24 m emitted from a 1630 nm laser.

Without loss of generality, we examined the one-dimensional intensity distribution of the beam profiles shown in Fig. S9n and Fig. S10n respectively. We cut the beam profiles along the vertical direction to obtain one-dimensional intensity distribution. In the following, we smooth and normalize the intensity distribution. The final results are shown in Fig. S11. It shows that both beam profiles in the form of first-order Bessel functions. Similar images can be seen in Fig 2 of Ref^1^ and Fig. 3 of Ref^7^. It can be seen from Fig. S8 to Fig. S10 that the profile of the light field has not changed much except for the contrast.


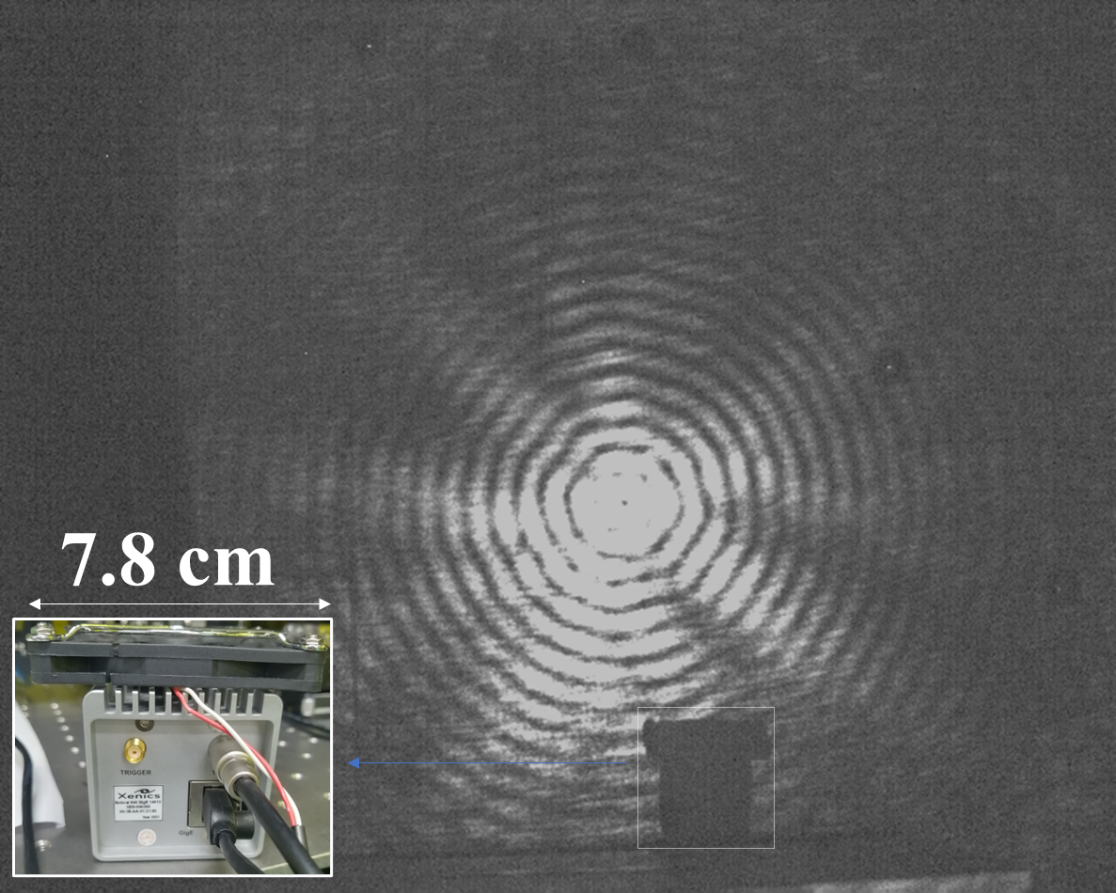


**Fig. S12. The complete profile of BGb at 10.24m.** The inset is the infrared camera (Bobcat 640 GigE) with a fan used in the experiment

Actually, all pictures in Fig. S8 –Fig. S10 have a scale printed in advance, which can visually read the size of the light spot. The spot diameter of the innermost ring is 0.41 cm, 1.54 cm and 2.45 cm at *z* = 1.55 m, 5.91 m and 10.24 m (distance to chip surface) respectively. It can be seen from the above pictures that the BGb generated by the integrated photonic chip propagates in a conical divergent manner. As shown in Fig. S12, we can clearly see that there are 17 concentric rings in BGb at 10.24 m. This shows that the Gaussian beam emitted by each grating arrays fills the entire space in Fig. S12 at a distance of 10.24 m.

Section 6. The method of measuring rotational speed by using BGb generated by integrated photonic chip.

The BGb profile from the chip can be descript by equation^14^ (S6.1) and the *C* is constant for a given *z*. This field can be regarded as the superposition of two beams with opposite topological charges (*l* = ±1) and different polarization states right (or left) handed circularly polarized (RHCP) (or LHCP) light:

(S6.1)

The Jones matrix of half wave plate (HWP) with an angle between the fast axis and the *x* axis of *α*, quarter wave plate (QWP) with *β* and polarization beam splitter (PBS) are:

$HWP_{\alpha}=\left(\begin{matrix} \cos2\alpha& \sin2\alpha\\ \sin2\alpha& -\cos2\alpha\end{matrix}\right)\begin{matrix} \end{matrix}QWP_{\beta}=\left(\begin{matrix} 1-i\cos2\beta& -i\sin2\beta\\ -i\sin2\beta& 1+i\cos2\beta\end{matrix}\right)\begin{matrix} \end{matrix}PBS=\left(\begin{matrix} 1 & 0 \\ 0 & 0 \end{matrix}\right)$ (S6.2)

When the light beam illuminates the surface of the rotating object, frequency shift will occur due to the rotating Doppler effects.

(S6.3)

Where *ω*_1_ = 2π (*f* - Δ*f*), *ω*_2_ = 2π (*f* + Δ*f*), and Δ*f* *=* *l*Ω/2π and the Ω is rotation of angular frequency. The signal captured by the Avalanche photodiodes is *I*(*t*) = $\vec{E}^{*}\vec{E}$, and the former is the transposed conjugate of the latter.

… (S6.4)

The above formula shows that the difference frequency of the echo has nothing to do with *β*. For a fixed *α* angle and topological charge *l*, the difference frequency is only related to the rotation speed of the object. Hence, the modulation frequency that can be detected by the oscilloscope is (S6.5), which is consistent with the previous reports.

$f_{\mathrm{mod}}=\left(\omega_{2}-\omega_{1}\right)/2\pi=l\Omega/\pi=\Omega/\pi$ (S6.5)

In fact, polarizer (HWP and PBS) here is very important for the whole experiment. It is proved below that the echo will not contain differential frequency information without polarizer. When the light from the chip directly shines on the surface of a rotating object：

(S6.6)

(S6.7)

The above formula shows that if BGb does not pass through the polarizer, there is no rotational speed information related to the rotary Doppler Effect in the intensity spectrum.

Section 7. The self-healing characteristic of the BGb in both the transverse and longitudinal directions.


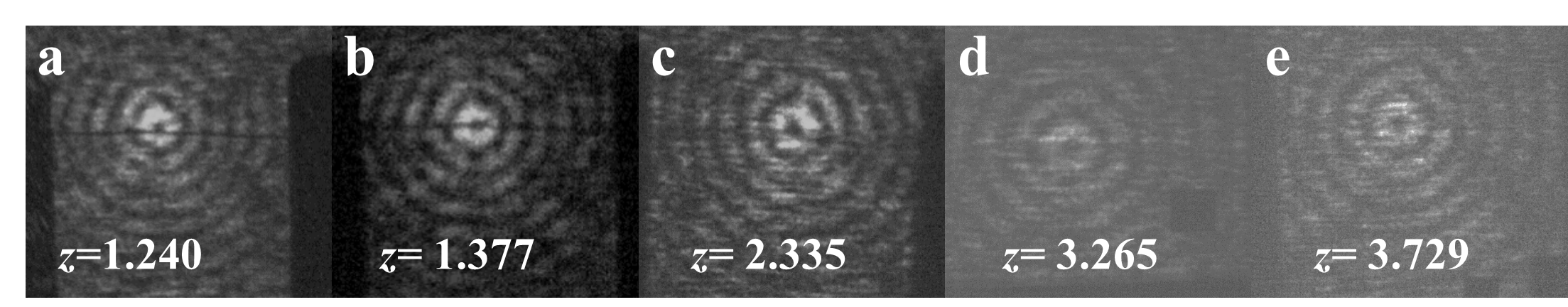


**Fig. S13. The change of the beam profile with the propagation distance after the Obstruction (Transverse).** **a** It is 1.24 m from the chip surface and 0.02 m from the obstacle, a cylindrical copper wire with a diameter of 0.36 mm. **b-e** 1.377 m, 2.335 m, 3.265 m, and 3.729 m, respectively, from the chip surface.



Section 8. Influence of sampling time of oscilloscope on measurement results of rotational speed.


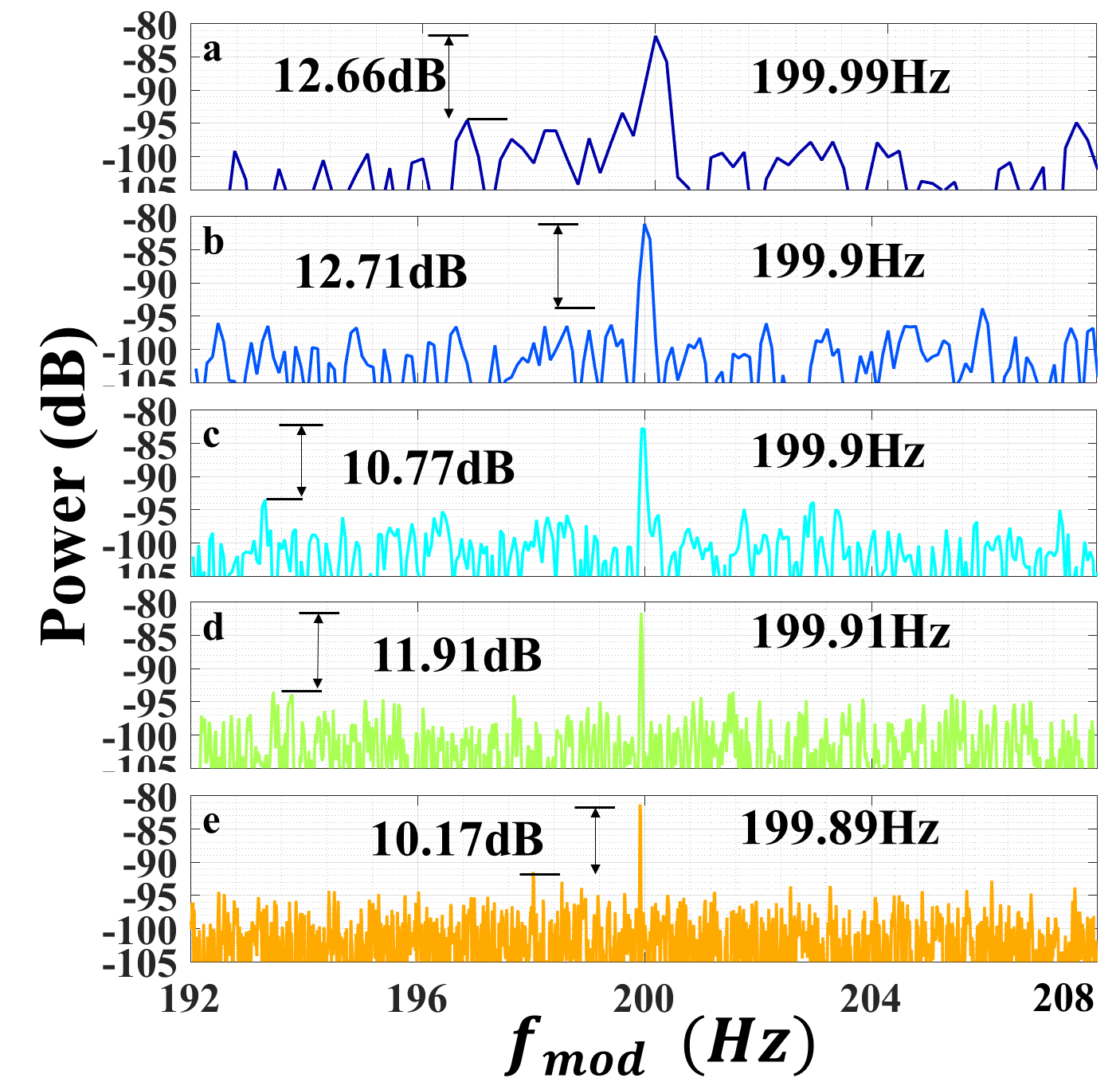


**Fig. S14. Rotating speed measurement results at different sampling times using BGb generated on the chip.** **a** is the result of the fast Fourier transform of the intensity information accumulated for 5 s, **b** 10 s, **c** 20 s, **d** 50 s, and **e** 100 s.

When the time is short, the frequency-domain bandwidth is large, and the sampling rate is low. It can be seen from Figure S14a that the data is sparse, and the peak width is relatively large. After more than 20 seconds, several times of time consumption could not have improved the measurement accuracy more effectively.

Section 9. Research on Rotational Speed Measurement Based on Rotational Doppler Effect in Recent Years.

**Table S3. Development of rotational speed measurement based on rotational Doppler effects.**

| Year | Light source  method | Min topological charge | Wavelength | Max rotational  speed | Max error |
| --- | --- | --- | --- | --- | --- |
| 2013^15^ | SLM | ±16 | 670 nm | 500 rad/s | 1954.5±4.9  0.251% |
| 2014^16^ | SLM | ±8 | white-light | <420 rad/s  (Fig. 4) | -- |
| 2016^17^ | RF OAM beam generation source | 1 | 20GHz | 50 rad/s | 0.67% |
| 2017^18^ | SLM | ±16 | 1.55 μm | <575 rad/s  (Fig. 5) | -- |
| 2020^19^ | SLM | ±16 or  +30, -2 | 632.8 nm | 4402 rad/s | < 5% |
| 2019^20^ | SLM | ±12 | 632.8 nm | 716.3 rad/s | < 2% |
| 2019^21^ | SLM | ±10 | 1.6 μm | 94.25 rad/s | < 10% |
| 2022^22^ | SLM | ±10 | 532 nm | 31.4 rad/s | < 4% |
| This Work | Integrated photonic chip | ±1 | 1.5-1.63 μm | 628 rad/s | 0.05% |

Supplementary video V1：

The longitudinal (perpendicular to the propagation direction) self-healing characteristic of the BGb. The copper wire with a diameter of 0.1 mm is 0.149 m away from the chip surface, and the light screen is 1.67m away from the chip surface. The optical wavelength used is 1525 nm.

Supplementary video V2.

The intensity distribution of the BGb generated by integrated photonic chip after passing through rotating polarizer.

Supplementary video V3.

Animation when measuring the speed of rotating objects (100 *r*/*s*). The shape of BGb generated on-chip on a disc with a diameter of 4.85 cm after HWP, PBS, QWP and mirror as Fig. 3

Supplementary video V4.

Animation of rotational speed measurement with an obstacle (100 *r*/*s*). The obstacle is a 2 mm diameter copper wire, corresponding to Fig 5b above each cell.

References

1 Chen, W. T. *et al.* Generation of wavelength-independent subwavelength Bessel beams using metasurfaces. *Light Sci Appl* **6**, e16259, doi:10.1038/lsa.2016.259 (2017).

2 Notaros, J., Poulton, C. V., Byrd, M. J., Raval, M. & Watts, M. R. Integrated optical phased arrays for quasi-Bessel-beam generation. *Opt Lett* **42**, 3510-3513, doi:10.1364/OL.42.003510 (2017).

3 Rao, A. S. & Samanta, G. K. On-axis intensity modulation-free segmented zero-order bessel beams with tunable ranges. *Opt Lett* **43**, 3029-3032, doi:10.1364/OL.43.003029 (2018).

4 Fan, Y. *et al.* 2D Waveguided Bessel Beam Generated Using Integrated Metasurface-Based Plasmonic Axicon. *ACS Appl Mater Interfaces* **12**, 21114-21119, doi:10.1021/acsami.0c03420 (2020).

5 Qu, M. J., Li, W. Y., Zeng, T., Su, J. X. & Song, W. L. 3D printed metasurface for generating a bessel beam with arbitrary focusing directions. *Opt Lett* **46**, 5441-5444, doi:10.1364/OL.440977 (2021).

6 Zhang, M. *et al.* optical element to generate zero-order quais-bessel beam with focal length. *Opt Lett* **47**, 553-556, doi:10.1364/OL.448852 (2022).

7 Reddy, I. V. A. K., Bertoncini, A. & Liberale, C. 3D-printed fiber-based zeroth- and high-order Bessel beam generator. *Optica* **9**, 645-651, doi:10.1364/optica.453839 (2022).

8 Cheng, J., Yang, Y., Fan, F., Wang, X. & Chang, S. Terahertz tight-focused Bessel beam generation and point-to-point focusing based on nonlocal diffraction engineering. *Opt Lett* **47**, 2879-2882, doi:10.1364/OL.460747 (2022).

9 Céspedes Vicente, O. & Caloz, C. Bessel beams: a unified and extended perspective. *Optica* **8**, doi:10.1364/optica.411887 (2021).

10 Gori, F., Guattari, G. & Padovani, C. BESSEL-GAUSS BEAMS. *Opt Commun* **64**, 491-495, doi:Doi 10.1016/0030-4018(87)90276-8 (1987).

11 Wu, G. a. W., Fei and Cai, Yangjian. Generation and self-healing of a radially polarized Bessel-Gauss beam. *Phys. Rev. A* **89**, 043807, doi:10.1103/PhysRevA.89.043807 (2014).

12 Parsa, S., Fallah, H. R., Ramezani, M. & Soltanolkotabi, M. Theoretical and experimental investigation of generating pulsed Bessel-Gauss beams by using an axicon-based resonator. *Appl Opt* **51**, 7475-7481, doi:10.1364/AO.51.007475 (2012).

13 Jordan, R. H. & Hall, D. G. Free-space azimuthal paraxial wave equation: the azimuthal Bessel-Gauss beam solution. *Opt Lett* **19**, 427-429, doi:10.1364/ol.19.000427 (1994).

14 Zhi, Z. H. *et al.* A Theoretical Description of Integrated OAM Beam Emitters Using Conical Wave Model. *Ieee Photonics Journal* **14**, 1-6, doi:10.1109/Jphot.2022.3149808 (2022).

15 Lavery, M. P., Speirits, F. C., Barnett, S. M. & Padgett, M. J. Detection of a spinning object using light's orbital angular momentum. *Science* **341**, 537-540, doi:10.1126/science.1239936 (2013).

16 Lavery, M. P. J., Barnett, S. M., Speirits, F. C. & Padgett, M. J. Observation of the rotational Doppler shift of a white-light, orbital-angular-momentum-carrying beam backscattered from a rotating body. *Optica* **1**, doi:10.1364/optica.1.000001 (2014).

17 Zhao, M. *et al.* Measurement of the rotational Doppler frequency shift of a spinning object using a radio frequency orbital angular momentum beam. *Opt Lett* **41**, 2549-2552, doi:10.1364/OL.41.002549 (2016).

18 Fu, S., Wang, T., Zhang, Z., Zhai, Y. & Gao, C. Non-diffractive Bessel-Gauss beams for the detection of rotating object free of obstructions. *Opt Express* **25**, 20098-20108, doi:10.1364/OE.25.020098 (2017).

19 Qiu, S. *et al.* Spinning object detection based on perfect optical vortex. *Opt Laser Eng* **124**, doi:10.1016/j.optlas-eng.2019.105842 (2020).

20 Qiu, S. *et al.* Detection of spinning objects at oblique light incidence using the optical rotational Doppler effect. *Opt Express* **27**, 24781-24792, doi:10.1364/OE.27.024781 (2019).

21 Zhai, Y., Fu, S., Yin, C., Zhou, H. & Gao, C. Detection of angular acceleration based on optical rotational Doppler effect. *Opt Express* **27**, 15518-15527, doi:10.1364/OE.27.015518 (2019).

22 Qiu, S., Ding, Y., Liu, T., Liu, Z. & Ren, Y. Rotational object detection at noncoaxial light incidence based on the rotational Doppler effect. *Optics Express* **30**, doi:10.1364/OE.461179 (2022).
